# The Bigger Picture: Why Oral Mucosa Heals Better Than Skin

**DOI:** 10.3390/biom11081165

**Published:** 2021-08-06

**Authors:** Maaike Waasdorp, Bastiaan P. Krom, Floris J. Bikker, Paul P. M. van Zuijlen, Frank B. Niessen, Susan Gibbs

**Affiliations:** 1 Department of Molecular Cell Biology and Immunology, Amsterdam Infection and Immunity Institute, Amsterdam University Medical Centers, Vrije Universiteit Amsterdam, De Boelelaan 1108, 1081 HZ Amsterdam, The Netherlands; s.gibbs@amsterdamumc.nl; 2 Department of Preventive Dentistry, Academic Center for Dentistry Amsterdam (ACTA), University of Amsterdam and Vrije Universiteit Amsterdam, 1081 HZ Amsterdam, The Netherlands; b.krom@acta.nl; 3 Department of Oral Biochemistry, Academic Center for Dentistry Amsterdam (ACTA), University of Amsterdam and Vrije Universiteit Amsterdam, 1081 HZ Amsterdam, The Netherlands; f.bikker@acta.nl; 4 Burn Centre, and Department of Plastic, Reconstructive and Hand Surgery, Red Cross Hospital, Vondellaan 13, 1942 LE Beverwijk, The Netherlands; p.vanzuijlen@amsterdamumc.nl; 5 Department of Plastic, Reconstructive and Hand Surgery, Amsterdam Movement Sciences, Amsterdam University Medical Centers, Vrije Universiteit Amsterdam, De Boelelaan 1117, 1007 MB Amsterdam, The Netherlands; fb.niessen@amsterdamumc.nl; 6 Department of Pediatric Surgery, Amsterdam University Medical Centers, Academic Medical Center, Meibergdreef 9, 1105 AZ Amsterdam, The Netherlands; 7 Department of Oral Cell Biology, Academic Center for Dentistry Amsterdam (ACTA), University of Amsterdam and Vrije Universiteit Amsterdam, 1081 HZ Amsterdam, The Netherlands

**Keywords:** wound healing, skin, oral, saliva, microbiome, scar, cytokines, growth factors

## Abstract

Wound healing is an essential process to restore tissue integrity after trauma. Large skin wounds such as burns often heal with hypertrophic scarring and contractures, resulting in disfigurements and reduced joint mobility. Such adverse healing outcomes are less common in the oral mucosa, which generally heals faster compared to skin. Several studies have identified differences between oral and skin wound healing. Most of these studies however focus only on a single stage of wound healing or a single cell type. The aim of this review is to provide an extensive overview of wound healing in skin versus oral mucosa during all stages of wound healing and including all cell types and molecules involved in the process and also taking into account environmental specific factors such as exposure to saliva and the microbiome. Next to intrinsic properties of resident cells and differential expression of cytokines and growth factors, multiple external factors have been identified that contribute to oral wound healing. It can be concluded that faster wound closure, the presence of saliva, a more rapid immune response, and increased extracellular matrix remodeling all contribute to the superior wound healing and reduced scar formation in oral mucosa, compared to skin.

## 1. Introduction

**Figure 1:**
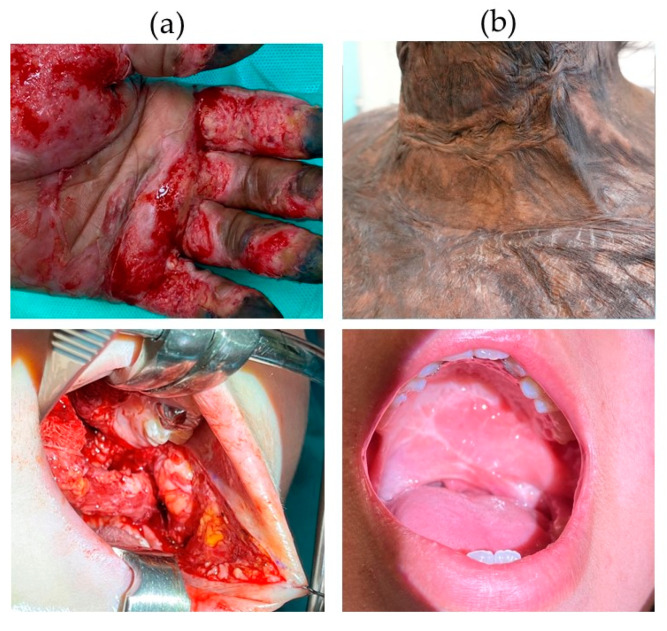
Comparison of human skin and oral mucosal wounds and scars. (**a**) Clinical example of a burn wound in skin (upper picture) and an oral wound from schisis corrective surgery (lower picture); (**b**) clinical example of a skin scar after burn injury (**upper picture**) and an oral scar after schisis corrective surgery (**lower picture**).

Wound healing is a tightly regulated process aiming to restore tissues upon damage. Irrespective of the type of wounded tissue, the process of wound healing follows four partially overlapping phases: hemostasis, inflammation, proliferation, and remodeling. Each stage specifically involves unique cell types and (signaling) molecules. Dysregulation at any phase, for example wound infection, may result in delayed wound healing, hypertrophic scar formation, and contractures [1]. Hypertrophic scars are red, raised, painful, and itchy. Contractures are very stiff and often cause reduced joint mobility, thus requiring multiple corrective surgeries. Up to 90% of (deep) burn wounds and 30% of surgical wounds heal with hypertrophic scarring [2,3]. Hypertrophic scar formation is associated with increased angiogenesis and a suppressed and delayed inflammatory response [4,5]. In general, wounds in the oral mucosa heal relatively fast and with little scarring, despite being continuously exposed to motion, tension, mechanical abrasion, and a great variety of microbes. In recent literature, it has been suggested that certain microbes may have a positive effect on wound healing, for example by training or alerting the immune system and thereby influencing the wound-healing cascade [6,7,8,9]. Similar to skin, complications during oral wound healing and underlying (autoimmune) diseases such as cicatricial pemphigoid, pemphigus vulgaris, or diabetes cause delayed wound healing and scarring in the oral mucosa [10,11]. External factors such as alcohol and cigarette smoke have been shown to impair oral wound healing as well [12]. Figure 1 illustrates clinical examples of human skin and oral wounds and healing outcome.

By understanding the mechanisms behind oral wound healing leading to acceptable scar formation, it may be possible to get insights into how to achieve reduced scarring of the skin or even complete tissue regeneration after wound healing. Several studies examined the differences between skin and oral wound healing that may explain the superior healing outcome in oral mucosa. Most of these studies however focus only on a single stage of wound healing or a single cell type and do not take into account the presence of saliva or the site-specific microbiome. The aim of this review is therefore to compare oral and skin wound healing during the entire healing process, including all cells participating in the healing process as well as the effect of environmental factors such as the presence of saliva and the oral microbiome.

## 2. The Intrinsic Differences between Skin and Oral Mucosa

**Figure 2:**
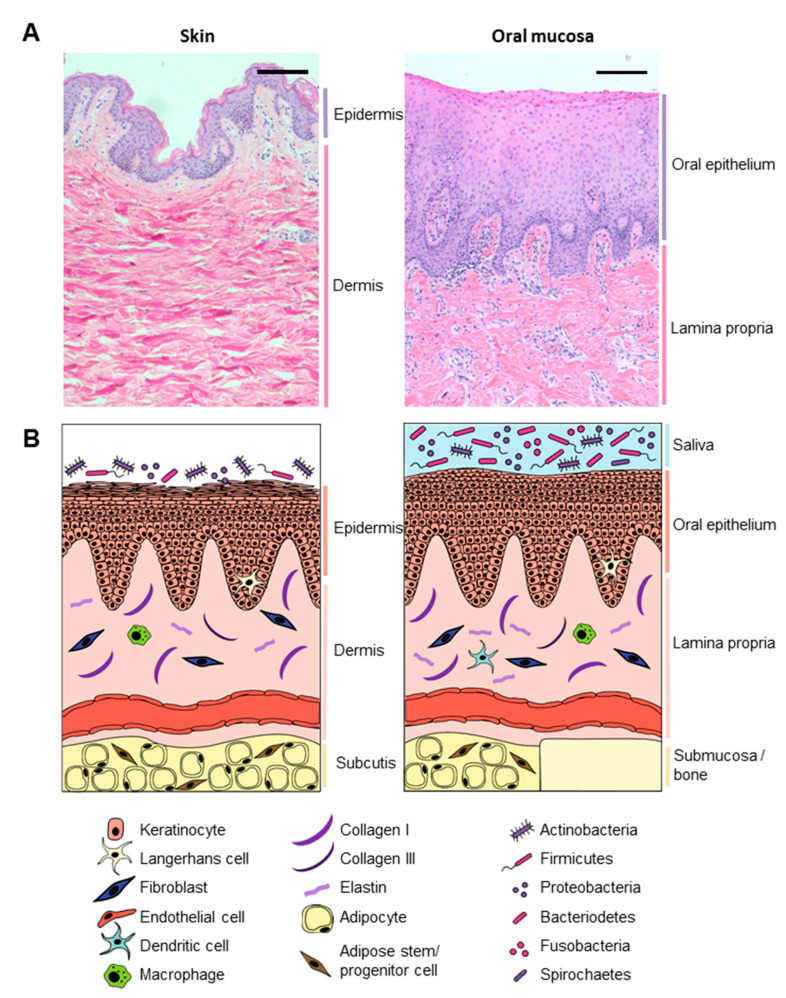
Comparing healthy skin and oral mucosa. (**A**) Histological comparison between healthy skin (**left**) and gingiva (**right**) tissue. Hematoxylin and eosin staining of 5 µm paraffin embedded tissue sections. Scale bar: 100 µm; (**B**) graphical illustration comparing skin (**left**) and oral mucosa (**right**).

When comparing the general tissue architecture, healthy skin and oral mucosa share many features, as well as have intrinsic histological differences, as illustrated in Figure 2. Both tissues comprise stratified epithelia, containing keratinocytes, melanocytes, Merkel cells, and Langerhans cells, which provide protection against body fluid loss, exposure to toxins, and microbial invasion [13,14]. Although skin and oral mucosa are generally alike in morphology and functions, there are differences already in homeostatic conditions that should be taken into account when comparing the healing process in both tissue types. For example, the oral epithelium is generally thicker compared to skin, as both palate and buccal mucosa consist of considerably more cell layers and a higher proliferation rate in the basal lamina compared to skin (20–30 versus 5–8 living cell layers, respectively) [15]. Whereas the epidermis is entirely keratinized, there is a clear differentiation within the oral cavity between the keratinized epithelium of the hard palate and of gingiva, which have to withstand the mechanical forces during mastication versus the nonkeratinized epithelium of the buccal mucosa that has the flexibility to stretch and withstand compression forces [14,16].

The stratified epithelium is supported by a subsurface layer of connective tissues (dermis for skin and lamina propria for oral mucosa) that contains fibroblasts, macrophages, mast cells, blood vessels, and nerve endings embedded in the extracellular matrix (ECM), that provides the epithelium with structural support and nutrients needed for continuous renewal [17]. Based on alpha-smooth muscle actin (α-SMA) and CD31 expression, more blood vessels are present in the oral mucosa (found in human palate tissue as well as in murine tongue tissue) compared to skin [18,19,20], although one study found no significant difference in the number of blood vessels at baseline in pig skin and gingiva based on laminin-1 expression [18,21]. Both dermis and lamina propria ECM mainly consist of collagen type I and type III (both in a ratio of about 5:1); although in nonkeratinized oral epithelia (e.g., buccal mucosa and soft palate), the ECM has a looser structure and contains more elastin compared to skin, hard palate, or gingiva [20,22]. In skin and buccal mucosa, the connective tissue layer is on top of an adipose tissue layer containing adipocytes and adipose stem and progenitor cells, whereas the lamina propria of the hard palate and gingiva is attached directly to bone via the mucoperiostium [14].

### The Microenvironment, Microbiome, and Saliva

Many of the intrinsic differences between skin and oral mucosa relate to the different environmental factors that the tissues are exposed to [15]. For example, the surface of the skin is exposed to air and constantly changing temperatures and humidity, whereas the oral mucosa is a continuously warm and wet environment, which makes an excellent habitat for a plethora of microbes. Moreover, the oral mucosa has to withstand heavy mechanical abrasion and is continuously exposed to foreign peptides, proteins, and antigens from food. The different substances in direct contact with skin and mucosa, such as sweat, oil, dry air, mucous, or saliva, all differ in pH, composition, and function and therefore differentially influence wound healing. For example, skin wound healing is accelerated in a moist environment resulting in faster re-epithelialization, angiogenesis, and maturation of the wound bed [23,24,25,26]. Not only does saliva naturally provide a wet environment in the oral cavity during wound healing, it also contains a myriad of peptides and proteins such as growth factors (epidermal growth factor or EGF, vascular endothelial growth factor or VEGF, and fibroblast growth factors or FGFs) and histatins, which stimulate wound healing [27,28,29]. Indeed, desalivated mice show reduced wound healing compared to sham-operated mice, both in skin and oral mucosa [30,31,32].

**Figure 3:**
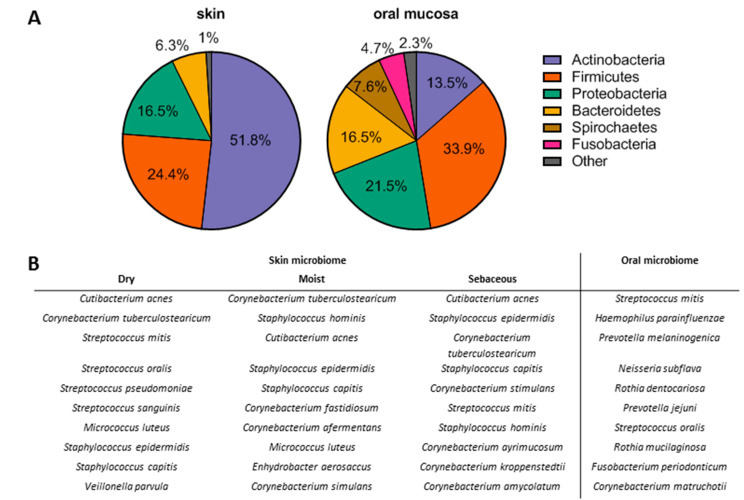
The microbiome in healthy skin and oral mucosa. (**A**) Dominating bacterial phyla in skin and oral mucosa; (**B**) most common bacterial species in skin and oral mucosa [33,37,40].

Next to having a direct effect on wound healing, environment-specific substances also create niches resulting in differential microbial composition and colonization of the different body surfaces. The total healthy oral cavity overall contains a higher number of microbes per surface area and, with currently around 700 unique species identified, has a greater diversity in microbial species (alpha diversity) compared to skin [7,33,34,35,36,37,38,39]. Although the dominating phyla are generally similar between skin and oral mucosa (actinobacteria, firmicutes, proteobacteria, and Bacteroidetes account for more than 90% of identified species), the skin microbiome is dominated by actinobacteria (50%), while in the oral cavity, the contribution of actinobacteria, firmicutes, proteobacteria, and Bacteroidetes is more equally distributed (Figure 3). *Cutibacterium acnes*, *Corynebacterium tuberculostearicum*, and *Staphylococcus epidermidis* are commonly found on skin, independent of the location (dry, wet, or sebaceous) [33,40].

Although wound infection caused by colonization of pathogenic microbes greatly delays wound healing (both in oral and skin), a healthy oral biofilm was found to result in an increased expression of antimicrobial peptides and improved barrier function in reconstituted human gingiva in vitro [41,42]. A positive effect of microbes on wound healing has been described to proceed via macrophage, dendritic cell and T-cell activation, and consequent cytokine production (TNF-α, IL-6, IL-10, and IL-17) which in turn stimulates stem cell proliferation [9,43].

Moreover, it has been reported that certain staphylococci can inhibit skin inflammation, suggesting that it may dampen the immune response during wound healing. Whereas *S. epidermidis* may benefit the host by dampening unwanted inflammation, this would create potentially favorable conditions for pathogenic staphylococci such as *Staphylococcus aureus*, to exploit suppression of keratinocyte activation as a mechanism of virulence [8]. A shift in the balance of host–microbe interaction leads to colonization of opportunistic Gram-positive bacteria at the patient’s skin wound site, followed by Gram-negative pathogens, such as *Pseudomonas aeruginosa*, and yeasts and fungi, such as *Candida albicans* [44]. In the oral cavity, saliva plays a major role in keeping the microbial balance in check [45]. Mucin 5B (MUC5B) and salivary agglutinin (SAG) are present in the protein films (pellicles) covering the enamel and epithelial surfaces and influence the microbial colonization of these surfaces [46]. In turn, SAG and mucin 7 (MUC7) are among the major bacteria—agglutinating factors in saliva, as shown by several studies reporting the binding of MUC7 and SAG to a variety of (oral) microorganisms (e.g., *Streptococcus sanguis*, *Streptococcus mitis*, *Streptococcus gordonii*, *Aggregatibacter actinomycetemcomitans*, *P. aeruginosa*, and *Escherichia coli*) [47,48,49,50]. Moreover, saliva contains many other antimicrobial peptides and enzymes such as defensins, histatins, cathelicidin (LL-37), lysozyme, lactoferrin, and lactoperoxidase, which synergistically eliminate microorganisms [45,46,51,52].

Taken together, microbes are beneficial both in homeostasis and during wound healing, as long as pathogenic species do not overgrow and colonize the tissue which is observed in large burn wounds, (oral) ulcers, or gingivitis [53,54]. The effect of microenvironmental factors such as saliva and microbes during the different phases of wound healing is discussed throughout this review. Although viruses and fungi have been shown to contribute to adverse wound healing, little information is available to compare skin with oral wound healing, and therefore, we focus on bacteria in the host–microbiome interaction during wound healing.

## 3. Differences between Skin and Oral Mucosa Wound Healing

**Figure 4:**
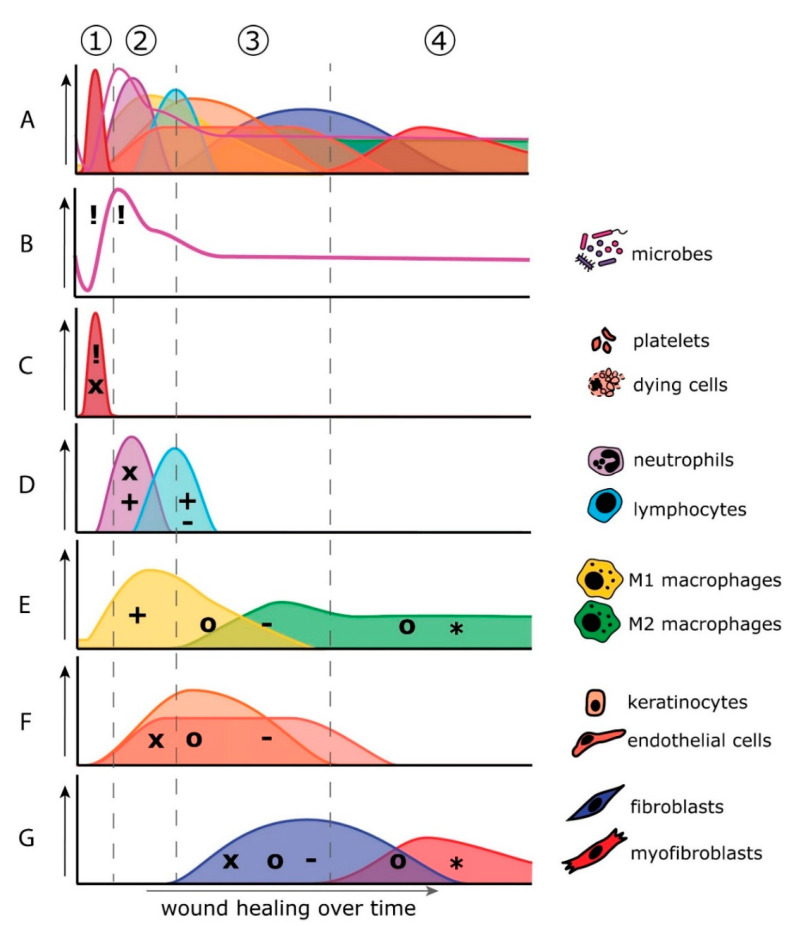
Overlapping phases of wound healing and relative contribution of cells, microbiome, and key molecules to wound healing over time. Wound healing is a well-orchestrated process that involves various cells and secreted factors. The separate phases of wound healing overlap in time and space and any change in one of the mediators (being cells or secreted molecules) affect healing outcome (**A**). Cell types and microbes involved in the stages of the wound healing process are shown individually (**B**–**G**) on the right side of the color coordinated graphs. (1) Hemostasis phase: minutes after wounding, the wound is quickly colonized by opportunistic and potentially pathogenic microorganisms releasing pathogen-associated molecular patterns (PAMPs; !) into the wound bed. Platelets and dying cells (**A**) release chemokines (x) and damage-associated molecular patterns (DAMPs; !) that attract immune cells (**C**). (2) Inflammation phase: minutes to hours after wounding, neutrophils are the first immune cell type to enter the wound bed. Neutrophils release chemokines (x) and proinflammatory cytokines (+) that attract and activate other immune cells such as lymphocytes and M1 macrophages (**D**,**E**). Macrophages contribute to proinflammatory cytokine secretion (+) and release growth factors (o) to stimulate tissue regeneration (**E**). (3) Proliferation phase: hours to days after wounding, keratinocytes, endothelial cells, and fibroblasts repopulate the granulation tissue via migration, proliferation, and differentiation in response to secreted chemokines (x) and growth factors (o) (**F**,**G**). Once the wound is closed, the resident cells secrete anti-inflammatory cytokines (-) to dampen the immune response (**F**,**G**). (4) Remodeling phase: weeks to months after wounding, (myo)fibroblasts (**G**), and M2 macrophages (**E**) that still reside in the wound bed remodel the extracellular matrix via the secretion of matrix proteins, matrix metalloproteinases (MMPs; *), and tissue inhibitors of metalloproteases (TIMPs; *).

**Figure 5:**
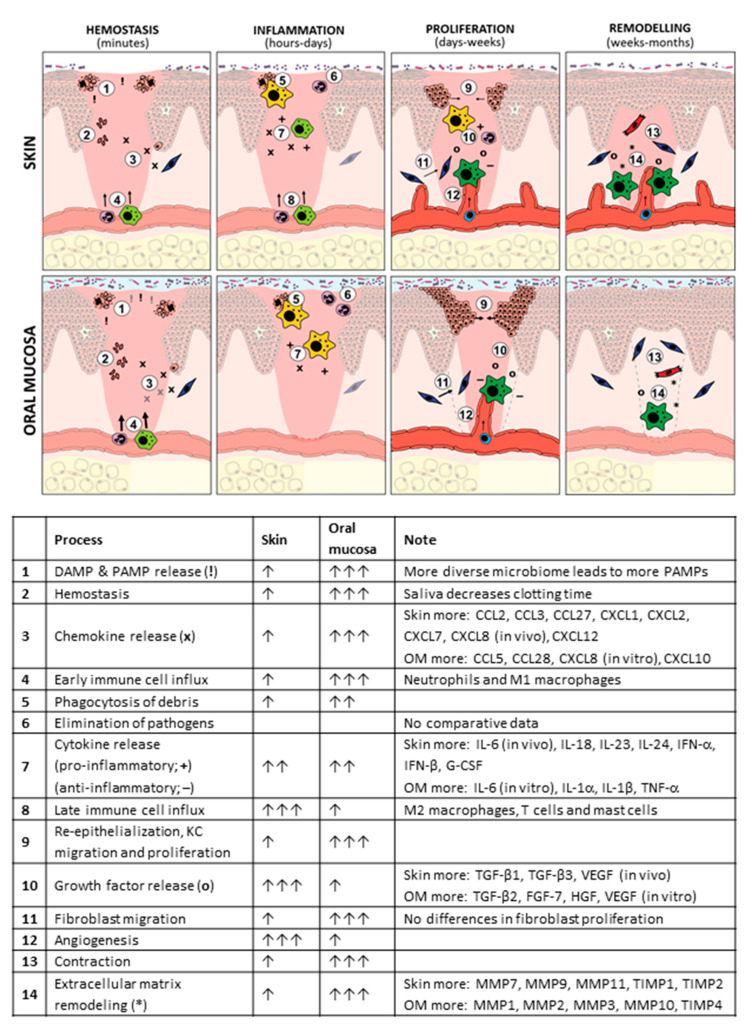
Overview of differences between oral and skin during wound healing. For more extensive information on all cells and molecules involved in skin versus oral wound healing and corresponding references, see Supplementary Tables S1–S3. DAMP (!), damage-associated molecular pattern; PAMP (!), pathogen-associated molecular pattern; OM, oral mucosa; IL, interleukin; KC, keratinocyte; TGF (o), transforming growth factor; VEGF (o), vascular endothelial growth factor; FGF (o), fibroblast growth factor; HGF (o), hepatocyte growth factor; MMP (*), matrix metalloproteinase; TIMP (*), tissue inhibitor of MMP.

Wound healing is a tightly regulated process that follows four partially overlapping phases: hemostasis, inflammation, proliferation, and tissue remodeling, with each phase involving different cell types and (signaling) molecules (Figure 4). An overview of all comparative data between skin and oral wound healing during each phase of wound healing is provided in Figure 5 and Supplementary Tables S1–S3 and discussed in the sections below.

### 3.1. Hemostasis Phase

Directly upon tissue damage and vascular rupture, the coagulation cascade is activated to prevent blood loss and provide a temporary seal to the wound (Figure 4; (1)). Although there are no data available that directly compares clotting times or platelet activation in skin and oral wounds, already in the 1930s, it was noticed that saliva reduces clotting time when added to blood samples [55]. This effect on coagulation could be attributed to the high levels of tissue factor (TF) found in extracellular vesicles in saliva [55,56]. Activated platelets and surviving keratinocytes and fibroblasts secrete chemokines, such as CXCL4, CXCL5, and CXCL8, to rapidly initiate the inflammatory phase by attracting immune cells to the wounded area [57,58]. Oral wounds in rat have been shown to express higher levels of platelet-derived growth factor (PDGF) as compared to skin wounds, which may indicate increased platelet activation in oral compared to skin wounds [59].

### 3.2. Inflammation Phase

The open-wound site provides an ideal niche for opportunistic pathogens to colonize, form biofilms, express virulence factors, and subsequently infect the host with related morbidity. The inflammation phase of wound healing is aimed at removing debris from the injured site and prevent infection by pathogens. Damage-associated molecular patterns (DAMPs) and pathogen-associated molecular patterns (PAMPs) in the wound bed trigger toll-like receptor (TLR), RAGE, and inflammasome signaling, leading to a cytokine and chemokine cascade released by resident cells that marks the onset of the inflammation phase, within hours after wounding (Figure 4; (2)). Neutrophils, monocytes, macrophages, mast cells, and T cells enter the wound bed in response to the chemokine gradients and drive the immune response against potential pathogenic invaders.

Many immune cells and inflammatory mediators also interact with resident cells throughout the course of wound healing and drive fibrotic responses [60,61]. Although low inflammation is associated with scarless fetal wound healing and increased inflammation is generally associated with (hypertrophic) scar formation, recent studies have also suggested that reduced inflammation in homeostasis and in the early wound-healing stages is a biomarker for hypertrophic scar formation [4,62,63]. In line, immunosuppressive drugs have been shown to impair wound healing after, for example, organ transplantation [64]. The inflammatory response influences the entire healing process, and its misbalance potentially leads to excessive tissue destruction and scar formation, wound infection, and delayed wound healing [65].

Interestingly, several commensals, such as *S. epidermidis*, have been shown to dampen inflammatory responses by inhibiting TLR3 signaling [8], whereas in hypoxic, lipid-rich environments, the commensal *C. acnes* breaks the immune tolerance via the release of short-chain fatty acids [66]. Host–microbial interactions may thus influence wound-healing outcome by manipulating immune cell function.

#### 3.2.1. Phagocytosis

After hemostasis, removal of debris starts with resident cells via phagocytosis. Already 4 h post injury, keratinocytes, fibroblasts, Langerhans cells, and resident macrophages start to phagocytose the resulting debris. Indeed, it was found that in rat skin, about 22% of the resident cells surrounding the injury contained phagocytosed material, compared to 35% of the cells in the oral mucosa (tongue) [67].

#### 3.2.2. Neutrophil Infiltration

Neutrophils are the first immune cells to enter the wounded area and play a crucial role in combatting microbial invasion via degranulation and phagocytosis [68]. Based on 2 mm excisional wounds in mice, infiltration of neutrophils starts as early as 4 h after injury and peaks after 24 h in both skin and oral mucosa [69]. The number of infiltrating neutrophils has been reported to be higher in skin during the whole course of wound healing, compared to the oral mucosa in mice [69]. By contrast, a human study (based on 3 mm excisional wounds) did not observe statistically different numbers of neutrophils during the first 6 days after wounding in skin and oral mucosa. However, the influx of neutrophils in the oral mucosa peaked 3 days after wounding followed by resolution, whereas in skin, the number of neutrophils kept rising up to 6 days after wounding, suggesting a more rapid influx of neutrophils in oral wound healing [36]. 

The more rapid influx of neutrophils found in oral wounds compared to skin might be the result of increased platelet activation and consequent release of chemokines such as CXCL4. Although high levels of TF in saliva have been shown to increase clotting time, the differences in platelet activation or level of CXCL4 have not yet been compared between skin and oral mucosa during wound healing. Another major neutrophil chemoattractant that is strongly increased in early wound healing is CXCL8 (also known as IL-8). Primary oral keratinocytes and fibroblasts produce more CXCL8 compared to primary skin keratinocytes and fibroblasts after stimulation with early inflammatory mediators such as TNF-α, IFN-γ, or UV irradiation [70,71,72]. By contrast, mice studies show that CXCL8 and other neutrophil-attracting chemokines (CCL3, CXCL1, CXCL2, CXCL5, and CXCL7) are higher expressed in skin wounds as compared to oral wounds. This may be due to species differences and is most likely due to the influx of immune cells into the wound bed and their significant contribution to the production of these chemokines and may be related to the prolonged influx of neutrophils in skin wounds [36,69,73].

Upon stimulation with cytokines such as TNF-α and IL-1β, keratinocytes increase expression of ICAM-1 [74], an adhesion molecule used by neutrophils to facilitate extravasation and migration into the wounded area. The increase in ICAM-1 expression is faster (upon IFN-γ stimulation) and greater (upon TNF-α stimulation) in oral keratinocytes compared to skin keratinocytes. Moreover, oral keratinocytes upregulate ICAM-1 expression upon IL-1α and IL-1β stimulation, while skin keratinocytes do not [74]. The more robust increase in ICAM-1 expression by oral keratinocytes and the higher production of neutrophil chemokines by oral resident cells may facilitate quicker influx of immune cells into the damaged area and thus contribute to the faster wound healing observed in the oral mucosa [70,71,72,74].

#### 3.2.3. Macrophage Infiltration

In addition to phagocytosing debris and killing microbial invaders by degranulation, neutrophils secrete chemokines such as CCL2 and CCL3 to attract monocytes into the wounded area [75]. Upon arrival, monocytes mature into macrophages, which contribute to phagocytosis of debris and replace the neutrophil population, becoming the predominant immune cell type in the wounded area. This takes approximately 2–4 days after wounding. Macrophages orchestrate the healing response via secretion of cytokines and growth factors that are initially proinflammatory (M1 phenotype), and in later stages of wound healing more anti-inflammatory and profibrotic (M2 phenotype) (Figure 4E).

In pigs, the number of macrophages peaks 3 days after wounding (both in oral and skin wounds), followed by resolution in oral wounds. In skin wounds, the number of macrophages remains high over time, resulting in significantly more macrophages in skin 14–60 days after wounding [21]. This correlates with increased levels of CCL2 and CCL3 in the later stages of wound healing in skin compared to oral in vivo [21,36,73]. Both skin keratinocytes and fibroblasts secrete more CCL2 upon stimulation with TNF-α and IFN-γ compared to their oral counterparts [71,76]. CCL3 expression is generally higher in skin and increases only 6 days after wounding [36,73]. So far, the differences between macrophages subtypes in skin and oral mucosa have not been compared.

#### 3.2.4. T Cells

Although T cells are known for their antimicrobial responses, wound healing can also occur in their absence. Nevertheless, a direct effect of T cells to wound healing has recently been recognized [77]. Infiltrated T cells produce an array of cytokines and growth factors that drives immune responses and wound healing [78,79,80]. 

In humans, it has been reported that 3 days after wounding, there is an influx of T cells in the oral mucosa, which resolves at day 6, while a steady but not significant influx of T cells was observed in skin wounds (up to 6 days after wounding), suggesting the influx of T cells is delayed in skin compared to oral mucosa [36]. In line with this hypothesis, Szpaderska et al. found more T cells in skin wounds in mice 7 days after wounding [69]. The T-cell chemokines CCL5 and CXCL10 were found at higher levels in oral wounds compared to skin, suggesting a bigger influx of Th1 in oral wounds. CCL27, a chemokine attracting Th22 and Tregs, was higher in skin compared to oral wounds [36,73,81,82]. In vitro studies using primary fibroblasts and keratinocytes showed that skin resident cells secrete more CCL20, CCL27, and CXCL12, whereas oral keratinocytes and fibroblasts secrete more CXCL10 and CCL28 [36,70,71,72,76,83]. This is in line with CCL27 and CCL28 being ligands for the same receptor with the former being skin specific and the later mucosa specific. Unfortunately, comparative data on T cell subsets and associated cytokines in skin and oral wound healing are not available to date, and therefore it has yet to be determined how this T-cell response influences the final quality of the scar. Interestingly, education of the immune system by commensal microbes can boost inflammation upon pathogen infection and also improve wound closure in a T-cell-dependent manner [6,84,85].

#### 3.2.5. Mast Cells

Other less recognized immune cells in the setting of wound healing are mast cells. Mast cells have been shown to stimulate proliferation, angiogenesis, and ECM deposition and remodeling via the release of cytokines and growth factors [86]. In pigs, mast cells have been shown to infiltrate both skin and oral wounds around 14 days post wounding, after which they remained in the skin wound bed but gradually disappeared in oral wounds. This resulted in significantly fewer mast cells in the oral wound bed 60 days after wounding [21].

#### 3.2.6. Cytokines

Cytokines in the wound bed heavily determine immune cell responses and wound healing outcome. Both infiltrating immune cells and resident cells produce and respond to cytokines. Cytokines have been classified as pro- or anti-inflammatory, or pro- or anti-fibrotic, although this black-and-white classification is hardly capturing the complexity of the cytokine system. Transcriptional analysis described in murine and human studies points toward a more proinflammatory cytokine profile in skin wounds, with increased expression of IL-6, IL-18, IL-23, IL-24, IFN-α, IFN-β, and G-CSF compared to oral wounds [36,69,73]. IL-18, IL-23, IL-24, and type 1 interferons have all been associated with scar formation and poor wound healing [87,88,89,90,91,92,93]. In oral wounds, IL-1α, IL-1β and TNF-α expression is higher compared to skin [36,69,73]. An extensive overview of differentially expressed cytokines in skin and oral wounds in vivo and expressed by skin and oral resident cells in vitro is provided in Supplementary Table S1.

Overall, the contribution of immune cells to wound healing appears to be prolonged in skin compared to oral wound healing. This trend of faster resolution of the inflammation in the oral wound compared to skin was also reflected in the number of T cells and monocytes, which were more frequently observed in skin compared to oral at the later stages of wound healing, which might be related to microbial clearance [21,36,69]. Alternatively, the quicker resolution of inflammation in oral wounds may simply be a result of the more rapid wound closure, since an intact epithelium is the main barrier against microbial invasion, the main trigger for inflammation [21,36,69,94,95]. As these studies are performed using different wound sizes and different species, they are not exactly comparable, especially regarding the time span of wound healing. However, all studies conclude that the inflammatory phase of wound healing is more robust and long lasting in skin compared to oral wounds [21,36,69,73]. A detailed overview of all studies and outcomes regarding the inflammatory phase in skin and oral wound healing is provided in Supplementary Table S1. A detailed comparison of the early inflammatory response, cytokines, immune cell subsets, and host–microbe interaction in skin versus oral wound healing is currently lacking but may provide more insight into this critical phase of wound healing and its implications for healing outcome.

### 3.3. Proliferation Phase

During the proliferation phase, which occurs hours to days after wounding, endothelial cells, fibroblasts, and epithelial cells migrate into the wound bed to regenerate the tissue (Figure 4; (3)). The highly vascularized and loosely organized granulation tissue made by fibroblasts and endothelial cells provides the wound bed with structural support and nutrition. Meanwhile, epithelial cells re-epithelialize the wound bed in a process of proliferation, migration, and differentiation [96].

#### 3.3.1. Angiogenesis

While angiogenesis is pivotal for rapid wound healing, increased numbers of blood vessels at later stages of wound healing are associated with hypertrophic scar formation [5]. Stagnation of the blood flow in the wounded area leads to hypoxia and triggers the angiogenic response. Cells in the hypoxic wound bed secrete various proangiogenic factors such as hypoxia-inducible factor 1α (HIF-1α) and VEGF to stimulate endothelial cell proliferation and migration, resulting in a large production of leaky vessels that provide the granulation tissue with nutrients and immune cells [97]. Although saliva is a rich source of VEGF, containing a significantly higher concentration compared to plasma (in healthy human volunteers) [27,98], and skin and oral keratinocytes produce similar amounts of VEGF upon hypoxia in vitro, higher levels of VEGF and HIF-1α were found in skin wound homogenates compared to oral wound homogenates in vivo [19,59,99]. In pigs, the number of vessels is increased 14 days after wounding both in oral and skin wounds, but the number of newly formed blood vessels is significantly higher in skin compared to oral wounds [21]. This increased vascular density during skin wound healing was also observed to be significantly higher in mice [19]. Interestingly, in both animal studies, the number of blood vessels returned to normal in the oral mucosa, while the vascular density was still increased in skin wounds up until 60 days after injury in pigs or 7 days in mice [19,21].

In conclusion, although both oral and skin wounds show increased angiogenesis in the wound bed (compared to uninjured tissue), angiogenesis and associated factors are higher in skin, where the number of newly formed blood vessels does not normalize after healing, as opposed to oral wounds. Increased vascular density has been associated with hypertrophic scar formation [5], in line with the increased scarring found in skin wounds in comparison to oral mucosal wounds. This reduced blood vessel regression in skin wounds may be the result of prolonged inflammation and subsequently higher energy demand, reduced CXCL10 expression, or delayed ECM and maturation found in skin wounds, as compared to oral wounds [36,100].

#### 3.3.2. Re-Epithelialization

Fast re-epithelialization of the wound bed is of vital importance to restore the barrier function of the skin and oral mucosa and to prevent infections and a delayed re-epithelialization is associated with adverse healing outcome [5,101]. Whereas air-exposed skin wounds are quickly covered with a scab under which the new epithelium is formed, oral wounds stay open during the entire wound-healing process and are continuously exposed to fresh saliva and the oral microbiome until complete re-epithelialization [21]. From macroscopic observations in humans, pigs, and rats, oral wounds close faster compared to skin wounds [21,36,59,95]. Based on histological evaluation, faster re-epithelialization is reported in 1–3 mm oral wounds compared to skin both in mice and humans, whereas in larger wounds in pigs no difference in re-epithelialization was observed between the two tissues (Supplementary Table S2) [21,36,67,69,95,96]. 

Migration and proliferation of keratinocytes are key events in re-epithelialization. In vitro assays comparing primary human keratinocytes isolated from the oral mucosa and skin show increased scratch closure with oral keratinocytes compared to skin keratinocytes due to increased proliferation and migration [16]. This was due to intrinsic properties of oral keratinocytes, as shown by the already increased expression of migration and proliferation-related genes in unwounded epithelial sheets, compared to skin [16]. Furthermore, oral fibroblasts produce more hepatocyte growth factor (HGF) and keratinocyte growth factor (KGF) (both known inducers of keratinocyte migration and proliferation [102]) compared to skin fibroblasts, which may additionally explain the increased rate of re-epithelialization of oral wounds compared to skin in vivo [103]. Another source of growth factors is saliva, which has been shown to contain thousands of bioactive proteins and peptides [27,98,104,105,106]. Indeed, whole saliva stimulates re-epithelialization of freeze wounds in reconstituted human gingiva and skin [107]. Histatins, a family of salivary peptides which is found only in higher primates, were identified as one of the most potent saliva-derived wound-healing peptides, and both histatin 1 and histatin 2 have been shown to stimulate migration of oral and skin keratinocytes in vitro [105,108,109].

Interestingly, commensal oral microbes such as *S. mitis* and *Streptococcus oralis* have also been shown to fasten wound closure in keratinocyte scratch assays, while pathogenic bacteria, e.g., *Porphyromonas gingivalis*, had an inhibitory effect on oral wound healing [42,110,111].

#### 3.3.3. Granulation Tissue Formation

While keratinocytes migrate and proliferate to restore the epithelial barrier, the formation of granulation tissue takes place to replace the wounded dermis. Fibroblasts and endothelial cells start secreting fibronectin to create a dense fibrillary network that enables cell migration into the wound bed. Fibronectin levels increase in both oral and skin wounds and peak 7–14 days after wounding in pigs. Interestingly, whereas fibronectin expression returns to normal in oral wounds, it remains high in skin up until 49 days after wounding in pigs [95]. 

The dense fibronectin network at the wound site is essential for the formation of a mature collagen I/III matrix [101]. During the initial stages of wound healing, collagen III is the major collagen type produced, followed by the secretion of collagen I to increase tissue strength and elasticity [101,112]. The number of cells producing procollagen I was significantly increased in both skin and oral wounds 14 days after wounding in pigs and remained elevated in skin up to 60 days after wounding, whereas it returned to homeostasis levels in oral mucosa within 35 days. Moreover, primary skin fibroblasts cultured either in three-dimensional (3D) sheets or collagen-based hydrogels showed increased expression of collagen I, collagen III, and elastin-1 compared to oral fibroblasts [83,113].

Next to the fibrous proteins that form a structural scaffold for the cells, the ECM contains numerous matricellular proteins (such as glycoproteins and proteoglycans) that promote matrix–cell interactions and modulate various cellular responses, such as cell adhesion, migration, and proliferation [112]. The expression of matricellular (glyco)proteins tenascin C, SPARC-1, THBS-2, and osteopontin has been wound to be increased in primary human skin fibroblasts compared to those isolated from the oral tissue, whereas in vivo, the level of tenascin C was higher in the oral mucosa compared to skin, both in the healthy tissue and during wound healing [83,95]. This difference may lie in the presence of other cell types, such as macrophages and keratinocytes, which contributes to fibronectin and tenascin C deposition in vivo [114].

In vitro studies comparing intrinsic properties of primary human fibroblasts from skin and oral mucosa show contrasting results. Whereas some studies reported increased proliferation in gingival fibroblasts compared to skin fibroblasts at 3–15 days of cell culture and increased life span associated with increased telomere length in oral fibroblasts [83,115], others showed no significant differences in proliferation between the two fibroblast populations [116,117,118,119]. One study also found increased migration in oral-isolated compared to skin-isolated fibroblasts in vitro [116], in contrast to another report, which reported similar migration rates [117]. Moreover, oral fibroblasts have been shown to adhere better to vitronectin and collagen IV [120], whereas another study reported better adherence of dermal fibroblasts to fibronectin, accompanied by increased cell spreading [121]. This differential adhesion which is accompanied by differential integrin expression may relate to differential migration of skin and oral fibroblasts into the wound bed [120,121]. Despite the different findings, all these in vitro studies seem to suggest the presence of a higher number of fibroblasts in oral wounds, compared to skin. Interestingly, however, when comparing skin and oral wounds, more fibroblasts are observed in the skin wound bed in vivo [21], suggesting that environmental factors rather than intrinsic differences between skin and oral fibroblasts determine cell migration and proliferation in vivo. For example, saliva was found to enhance fibroblast migration in vitro [107]. Several growth factors that are known to stimulate fibroblasts migration and proliferation, such as FGF2 or bFGF and EGF, are present in saliva, and one study in rats reported increased levels of FGF2 and EGF in oral wounds compared to skin [59]. By contrast, no difference in FGF2 was found between oral and skin wounds in mice [19] and also primary human oral and skin fibroblasts express similar levels of FGF2 [83]. Moreover, the levels of FGF2 in human saliva is expected to be too low to have a biologically significant effect [27]. Other peptides in saliva, for example histatin 1 and histatin 2, do show promising effects in stimulating fibroblast migration [109]. 

Another factor that plays a major role in fibroblast proliferation is TGF-β, which is mainly secreted by immune cells such as macrophages and T cells. Three isoforms of TGF- β are described: TGF-β1, TGF-β2, and TGF-β3. Whereas TGF-β1 is associated with scar formation, TGF-β3 is more associated with scarless fetal wound healing [122]. At baseline, primary human skin fibroblasts produce more TGF-β1 and TGF-β3 and less TGF-β2 compared to oral fibroblasts [83]. During wound healing in mice, higher levels of TGF-β1 and lower or similar levels of TGF-β3 were found in skin compared to oral wounds [69,96]. Contradictorily, no difference in TGF-β1 was found in porcine oral versus skin wounds, while TGF-β3 levels were higher in skin [123]. Interestingly, TGF-β has been reported to exert differential effects on skin and oral fibroblasts. While Lee et al. observed no increased proliferation in either skin of oral fibroblast cultures upon TGF-β stimulation, Meran et al. showed that TGF-β stimulation has opposite effects on skin and oral fibroblasts: TGF-β stimulation induced proliferation in skin fibroblasts in a HAS2-dependent manner, whereas it reduced proliferation in oral fibroblasts [124,125]. A detailed overview of all growth factors found in skin and oral wounds in vivo and produced by skin and oral resident cells in vitro is provided in Supplementary Table S2.

In conclusion, faster re-epithelialization, restoration of the vascular density and reduced ECM production are characteristic of oral wound healing during the proliferation phase. Both intrinsic properties of oral cells (i.e., keratinocyte proliferation and migration) and presence of saliva (via histatin 1 and histatin 2) contribute to these favorable wound-healing conditions.

### 3.4. Remodeling Phase

Once the wound is covered with a new epithelium and the host–microbial balance is restored, inflammation is quenched by anti-inflammatory cytokines. Macrophages switch from a proinflammatory M1 to a proresolution M2 phenotype and secrete growth factors, matrix metalloproteinases (MMPs), and tissue inhibitors of metalloproteinases (TIMPs) to stimulate (myo)fibroblast-mediated remodeling of the ECM. This final remodeling phase of wound healing may take weeks to months, resulting in scar tissue that may gradually resolve eventually, although the tissue may never regain its original tensile strength (Figure 4; (4)).

Based on comparative studies in humans, pigs, and mice, it has become evident that oral wounds heal with less scarring compared to skin wounds [21,95,123]. Oral wounds show less contraction and better restoration of the tissue architecture in terms of collagen structure and presence of rete ridges. Extracellular matrix remodeling is of pivotal importance for the final scar quality [4]. In healthy skin, collagen fibers are thicker compared to oral mucosa, and in vitro cultures fibroblasts from skin show higher collagen 1 and 3 expression compared to oral fibroblasts [20,83]. During wound healing, collagen bundles are much thinner and randomly organized in both skin and oral mucosa. In pigs, already 14 days after wounding, collagen density and maturity in the wound area resembles unwounded tissue in the oral mucosa, whereas in skin wounds the collagen fibers remain thin and loosely organized and oriented perpendicular to the wound edges up to 49 days after wounding [21]. A detailed overview of ECM molecules in skin and oral wounds in vivo and produced by skin and oral fibroblasts in vitro is provided in Supplementary Table S3.

The diminished collagen reorganization in skin may be linked to the reduced normalization of vasculature in skin wounds, as collagen reorganization leads to vascular degradation [100,126]. However, tissue contraction, secretion of MMPs, their related inhibitors, and deposition of ECM and matricellular proteins play a role in tissue remodeling, scar formation, and the final quality of the (scar) tissue as well. Initial wound contraction may be an effective way to decrease wound size and thereby reduce the risk of infections. However, when considering final scar quality, contraction is undesirable, as it results in stiffer scar tissue, reduced tensile strength, and potentially results in movement restriction due to contractures. Many studies have compared skin and oral fibroblast in their ability to induce collagen gel contraction or remodeling in vitro. In all of these studies, increased collagen hydrogel contraction was observed with oral fibroblasts [103,117,127,128,129]. α-SMA expression, as a measure for myofibroblast conversion, is generally linked to collagen gel contraction. Indeed, the percentage of α-SMA+ (myo)fibroblasts isolated from (healthy) oral mucosa is higher than in skin [117]. In addition, upon wounding in vivo, more myofibroblasts have been observed in the oral mucosa compared to skin, both at early (1–2 weeks [130]) and late time points (8 weeks [21]) after wounding. Contrarily, other studies reported higher α-SMA expression in skin fibroblasts compared to oral fibroblasts [103,127], despite increased collagen hydrogel contraction by oral fibroblasts. In these studies, the increased contraction by oral fibroblasts was attributed to increased MMP3 expression, resulting in increased cell migration-induced contraction, rather than α-SMA-mediated contraction [127]. The mechanisms by which contraction is induced in skin and oral wounds in vivo and the consequent impact on final scar quality remain unclear.

While fibroblasts are the main cell type involved in the remodeling phase of wound healing, their function is very much dependent on the production of cytokines by macrophages. While macrophages still reside in the wounded area up to 60 days after wounding in skin, the number of macrophages in oral wounds gradually decreases after peaking 14 days post wounding [18]. Macrophages secrete an array of cytokines and growth factors (e.g., TGF-β), that stimulate the transition of fibroblasts to myofibroblasts and their consequent production of ECM molecules [131]. In line with increased numbers of macrophages in skin wounds, increased levels of TGF-β1 and phosphorylated SMAD3 (pSMAD3; a marker for TGF-β pathway activation) are measured in skin wounds in mice [96]. Moreover, when comparing oral and skin fibroblasts in 3D cultures, skin fibroblasts produce more TGF-β1 and pSMAD3 [83].

Next to its general role in regaining tissue strength via MMPs and TIMPs, ECM remodeling directly affects wound healing, by for example, inducing vessel regression, stimulating cell migration and the release of inactive growth factors into the wound bed. MMPs can inactivate growth factors, chemokines, and cytokines via proteolytic cleavage and thus affect inflammatory and fibrotic responses. Indeed MMP2, whose active form was found to be increased in oral fibroblasts cultures, inhibits macrophage and T-cell chemotaxis (via CCL7 cleavage resulting in CCR1/2/3 antagonist activity), while MMP9, which is more expressed in skin-equivalent cultures, can cleave CXCL8, resulting in a more potent product and amplified neutrophil influx [132,133,134]. In general, low MMP or high TIMP expression is associated with fibrosis and scar formation, whereas high MMP expression is associated with chronic wounds and periodontal disease [135]. Interestingly, no comparison of MMPs and TIMPs in oral and skin wounds has been done so far. In primary human fibroblast cultures, the total MMP expression is lower in skin compared to the oral mucosa [83]. A detailed overview of MMP and TIMPs produced by skin and oral resident cells in vitro is provided in Supplementary Table S3. Whereas expression of MMP1, MMP2, MMP3, and MMP10 was higher in oral compared to skin fibroblasts, MMP7, MMP9, and MMP11 were more expressed by skin fibroblasts [83,128,136]. Interestingly, whereas overexpression of MMP7 and MMP9 is associated with delayed wound healing, MMP2 and MMP3 play an important role in keratinocyte migration which may therefore further explain the overall faster and scarless oral wound healing [137,138,139,140].

## 4. The Bigger Picture: When Intrinsic, Local, Systemic, and External Factors Come Together

When taking all differences between skin and oral mucosa into account, it is clear that a delicate balance exists in wound healing with the scales being tipped toward faster healing and better scar quality for oral mucosa. Many in vitro studies have shown intrinsic differences between cells isolated from skin and oral tissue. For example, oral keratinocytes showed increased proliferation and migration, and differential cytokine, chemokine, growth factor, and MMP expression have been shown for both oral- and skin-derived keratinocytes and fibroblasts. Although these studies provide detailed information and may partially explain the differential wound healing of skin and oral mucosa, they also lack complexity and therefore scarcely reflect in vivo wound healing. More advanced organotypic coculture models may provide missing links between the complex in vivo wound-healing processes and the details that in vitro systems provide. Indeed, differences in cell behavior may arise in cocultures in a more physiological model. For example, whereas oral fibroblasts secrete higher levels of MMP9 compared to skin fibroblasts, organotypic cocultures revealed that secretion of active MMP9 becomes higher in skin when fibroblasts are cultured together with epidermal keratinocytes [136].

Apart from intrinsic local and systemic factors, external factors such as humidity, saliva, mechanical tension and microbial burden, and ecology have been shown to contribute to wound healing and influence healing outcome. Interestingly, both beneficial and detrimental effects have been attributed to host–microbial interactions during wound healing. Studies using germ-free mice have shown that priming the skin with commensal microbes prior to wounding improves wound closure via Th17-mediated cytokine release [6]. In line with the hypothesis that oral tissue is primed toward a more inflammatory state by the continuous microbial exposure, ex vivo gingiva biopsies secrete higher amounts of proinflammatory cytokines (i.e., IL-6, IL-8, IL-1β, IL-10, and TNF-α) as compared to skin biopsies [141]. In contrast, sterile cultured reconstructed human skin and gingiva without microbes or immune cells secrete similar levels of abovementioned cytokines [71]. Exposure of sterile cultured reconstructed human gingiva to a commensal biofilm again stimulates IL-6 and IL-8 release [142].

From the microbial perspective, the transition from a dry skin environment to a moist and protein-rich wound environment probably induces extensive adaptation of the skin microbiome, whereas the oral microbiome that is already exposed to a moist and protein-rich environment during homeostasis possibly remains much more stable upon wounding. Consequently, the resident host immune cells may be better primed toward the oral wound microbiome, resulting in a more efficient and rapid immune response in oral as compared to in skin wounds. However, from the information currently available, it cannot be established whether differences between the oral and skin microbiome contribute to the superior wound healing in the oral mucosa, and whereas some bacterial species are described, very little information on the role of fungi such as Candida in wound healing is available [110,143,144]. Current advances in in vitro host–microbial coculture systems that combine complex host models with extended exposure to multispecies microbial communities may provide a useful tool in studying the complex host–microbial interaction during wound healing [41,145].

The ongoing inflammatory response and its associated cytokine profile are crucial throughout the wound-healing process. Especially in the later stages of wound healing, macrophage and T-cell differentiation depend on cytokines inside the wound bed and both influence fibroblast function, ECM remodeling, and scar formation [146,147]. Indeed, macrophages develop into M1 phenotype upon IFN-γ and LPS stimulation, whereas TGF-β, IL-4, and IL-10 are classical inducers of the anti-inflammatory M2 phenotype. M1 macrophages contribute to inflammation via the secretion of inflammatory cytokines (such as IL-1β, IL-6, and TNF-α), whereas M2 macrophages secrete large amounts of growth factors (such as TGF-β) that contribute to wound closure, angiogenesis, and ECM deposition [146]. Similarly, Th1 cells (and their innate counterpart ILC1) play an important role in clearing the wound from pathogens and debris, while Th2 and ILC2 dampen the immune responses and initiate regeneration [80,148]. Whereas the destructive type 1 response might harm the tissue when active for too long, excess accumulation of M2 and Th2 cells in later stages of wound healing is associated with (hypertrophic) scar formation [4,149]. Th17 cells have been shown to contribute to the host–microbiome interaction, which is particularly interesting to compare between oral and skin wound healing, considering the difference in microbial load between the two tissues. However, a comprehensive comparison of the cytokine network and associated macrophage phenotypes and T-cell subsets in oral and skin wound healing has not been described in literature so far.

In conclusion, faster wound closure, presence of saliva, more rapid immune response, increased anti-inflammatory cytokine release, MMP-mediated cleavage of chemokines, and ECM remodeling all contribute to the superior wound healing and reduced scar formation in oral mucosa, as compared to skin.

## Supplementary Materials

The following are available online at https://www.mdpi.com/article/10.3390/biom11081165/s1, Table S1: Direct comparison between skin and oral wound healing in processes involved in the inflammatory phase, Table S2: Direct comparison between skin and oral wound healing in processes involved in the proliferation phase, Table S3: Direct comparison between skin and oral wound healing in processes involved in the remodeling phase.
