# Supplementary material for: The Bigger Picture: Why Oral Mucosa Heals Better Than Skin
Source: Biomolecules. 2021 Aug 6;11(8):1165. doi: 10.3390/biom11081165 (PMC8394648; doi:10.3390/biom11081165)
Supplement: Supplementary file 1 [file biomolecules-11-01165-s001.zip › biomolecules-1277034-supplementary.pdf]

Supplementary

The Bigger Picture: Why Oral Mucosa Heals Better Than Skin

Maaike Waasdorp [1,*], Bastiaan P. Krom [2], Floris J. Bikker [3], Paul P. M. van Zuijlen [4,5,6], Frank B. Niessen [5], and Susan Gibbs [1,7]

[1] Department of Molecular Cell Biology and Immunology, Amsterdam Infection and Immunity Institute, Amsterdam University Medical Centers, location: Vrije Universiteit Amsterdam, , De Boelelaan 1108, 1081 HZ Amsterdam, The Netherlands; s.gibbs@amsterdamumc.nl
[2] Department of Preventive Dentistry, Academic Center for Dentistry Amsterdam (ACTA), University of Amsterdam and Vrije Universiteit Amsterdam, Amsterdam, The Netherlands; b.krom@acta.nl
[3] Department of Oral Biochemistry, Academic Center for Dentistry Amsterdam (ACTA), University of Amsterdam and Vrije Universiteit Amsterdam, Amsterdam, The Netherlands; f.bikker@acta.nl
[4] Burn Centre, and Department of Plastic, Reconstructive and Hand Surgery, Red Cross Hospital, Vondellaan 13, 1942 LE Beverwijk, The Netherlands; p.vanzuijlen@amsterdamumc.nl
[5] Department of Plastic, Reconstructive and Hand Surgery, Amsterdam Movement Sciences, Amsterdam University Medical Centers, location Vrije Universiteit Amsterdam, De Boelelaan 1117, 1007 MB Amsterdam, The Netherlands; fb.niessen@amsterdamumc.nl
[6] Department of Pediatric Surgery, Amsterdam University Medical Centers, location Academic Medical Center, Meibergdreef 9, 1105 AZ Amsterdam, The Netherlands
[7] Department of Oral Cell Biology, Academic Center for Dentistry Amsterdam (ACTA), University of Amsterdam and Vrije Universiteit Amsterdam, Amsterdam, The Netherlands
* Correspondence: m.waasdorp@amsterdamumc.nl

Citation: Waasdorp, M.; Krom, B.P.; Bikker, F.J.; van Zuijlen, P.P.M.; Niessen, F.B.; Gibbs S. The Bigger Picture: Why Oral Mucosa Heals Better Than Skin. *Biomolecules 2021, 11,* 1165. https://doi.org/10.3390/biom11081165

Academic Editors: Ryan Moseley, Robert Steadman and Adam C. Midgley

Received: 11 June 2021
Accepted: 2 August 2021
Published: 6 August 2021

Publisher's Note: MDPI stays neutral with regard to jurisdictional claims in published maps and institutional affiliations.

Copyright: © 2021 by the authors. Submitted for possible open access publication under the terms and conditions of the Creative Commons Attribution (CC BY) license (http://creativecommons.org/licenses/by/4.0/).

Table S1: Direct comparison between skin and oral wound healing in processes involved in the inflammatory phase

**Inflammation phase**	**model**			**difference**	**(ref)**
**(2-72 hours)**	**species**	**stimulus**	**Time** (h)		
**Immune cells**					
neutrophils (MPO)	Humans<br>Mice	3 mm biopsy<br>1 mm biopsy	24-72 hours<br>3-6 days<br>2, 4, 12, 24, 48, 72 hours	More in oral<br>More in skin<br>More in skin	[36]<br>[69]
Macrophages	Humans<br>Pigs<br><br>Mice	3 mm biopsy<br>15x27 mm excision<br>1 mm biopsy	24-72 hours<br>72 hours<br>14-60 days<br>2, 4, 12, 24, 48, 72 hours	More in skin<br>More in oral<br>More in skin<br>More in skin	[36]<br>[21]<br>[21]<br>[69]
T lymphocytes (CD3)	Humans<br>Mice	3 mm biopsy<br>1 mm biopsy	24-72 hours<br>7 days	More in oral<br>More in skin	[36]<br>[69]
Mast cells	Rat	15x27 mm excision	72 hours	More in skin	[21]
**Chemokines *in vivo***					
CCL2 (MCP-1)	Human	3mm biopsy	24-72 hours	More in skin	[36]
CCL3 (MIP-1a)	Human<br>Mice	3mm biopsy<br>1mm biopsy	24-72 hours<br>0 ,6 ,12, 24, 72 hours	More in skin<br>More in skin	[36]<br>[73]
CCL5 (RANTES)	Mice	1mm biopsy	0 ,6 ,12, 24, 72 hours	More in oral	[73]
CCL20	Mice	1mm biopsy	0 ,6 ,12, 24, 72 hours	No difference	[73]
CCL27	Human	3mm biopsy	1, 3, 6 days	More in skin*	[36]
CXCL1 (GRO1/KC)	Human	3mm biopsy	72 hours	More in skin	[36]
CXCL2 (MIP-2a)	Human	3mm biopsy	24-72 hours	More in skin	[36]
CXCL5 (ENA-78)	Human	3mm biopsy	24-72 hours	More in skin	[36]
CXCL7 (NAP-2)	Mice	1mm biopsy	0, 6, 12, 24, 72 hours	More in skin	[73]
CXCL8 (IL-8)	Human<br>Mice	3mm biopsy<br>1mm biopsy	24-72 hours<br>2, 4, 12, 24, 48, 72 hours	More in skin<br>More in skin	[36]<br>[69]
CXCL10	Human<br>Mice	3mm biopsy<br>1mm biopsy	1-2-6 days<br>2, 4, 12, 24, 48, 72 hours	More in oral*<br>More in skin*	[36]<br>[73]

	**model**			**difference**	**(ref)**
**Chemokines *in vitro***	**species**	**stimulus**	**Time** (h)		
CCL2 (MCP-1)	human tissue equivalent<br><br>primary human keratinocytes<br><br>primary human fibroblasts	TNF-α<br>UVB<br>TNF-α/ IFN-γ<br><br>TNF-α	24 hours<br>24 hours<br>8 hours<br>24-72 hours<br>24 hours	More in skin<br>No difference<br>More in oral<br>More in skin<br>More in skin	[71]<br>[70]<br>[76]<br>[76]<br>[71]
CCL3 (MIP-1a)	primary human keratinocytes	TNF-α/ IFN-γ/ IL-1α/IL-4/ TGF-β	2, 4, 12, 24, 48, 72 hours	No difference	[72]
CCL5 (RANTES)	primary human keratinocytes<br>primary human fibroblasts<br>human tissue equivalent	TNF-α/ IFN-γ<br>TNF-α<br>TNF-α<br>UVB	24 hours<br>24 hours<br>24 hours	No difference<br>No difference<br>No difference<br>No difference	[72]<br>[71]<br>[71]<br>[70]
CCL20	primary human fibroblasts<br>human tissue equivalent	TNF-α<br>TNF-α	24 hours<br>24 hours	No difference<br>More in skin*	[71]<br>[71]
CCL27	primary human keratinocytes<br>human tissue equivalent	TNF-α<br>Baseline	24 hours<br>24 hours	More in skin<br>More in skin	[71]<br>[71]
CCL28	primary human keratinocytes	TNF-α	24 hours	More in oral	[71]
CXCL8 (IL-8)	primary human fibroblasts<br>primary human keratinocytes<br>human tissue equivalent	TNF-α<br>TNF-α/ IFN-γ<br>TNF-α<br>UVB	24 hours<br>2, 4, 12, 24, 48, 72 hours<br>24 hours<br>24 hours	Same/oral?<br>More in oral<br>No difference<br>More in oral	[71]<br>[72]<br>[71]<br>[70]
CXCL10	Human tissue equivalent	UVB	24 hours	More in oral	[70]
CXCL12 (SDF-1)	human tissue equivalent<br>primary human fibroblasts	TNF-α<br>TNF-α<br>baseline	24 hours<br>24 hours<br>7 days	More in skin<br>More in skin<br>More in skin	[71]<br>[71]<br>[83]
ICAM-1	primary human oral/skin keratinocytes	TNF-α/ IFN-γ	24 hours	More in oral	[74]
**Cytokines *in vivo***					
IL-6	Human<br>Mice	3mm biopsy<br>1mm biopsy	24-72 hours<br>2, 4, 12, 24, 48, 72 hours	More in skin*<br>More in skin	[36]<br>[69]
IL-1α	Human	3mm biopsy	24-72 hours	More in oral*	[36]

IL-1β	Human	3mm biopsy	24-72 hours	More in oral (absolute)<br>More in skin (increase)	[36]
IL-10	Mice	1 mm biopsy	2, 4, 12, 24, 48, 72 hours	No difference	[69]
IL-23	Mice	1 mm biopsy	0, 6, 12, 24, 48 hours	More in skin*	[73]
IL-24	Mice	1 mm biopsy	0, 6, 12, 24, 48 hours	More in skin*	[73]
IFN-α/β	Mice	1 mm biopsy	0, 6, 12, 24, 48 hours	More in skin*	[73]
G-CSF	Mice	1 mm biopsy	0, 6, 12, 24, 48 hours	More in skin*	[73]
**Cytokines *in vitro***					
IL-6	human tissue equivalent<br><br>Primary human keratinocytes<br><br><br><br><br><br>Primary human fibroblasts	TNF-α<br>UVB<br>IL-1β<br><br>IFN-γ<br>TNF-α<br>IL-4<br>TGF-β<br>CXCL8<br>TNF-α	24 hours<br>24 hours<br>0, 6, 12, 24, 48 hours<br>24 hours<br>24 hours<br>24 hours<br>24 hours<br>24 hours<br>24 hours<br>24 hours	No difference<br>More in oral<br>More in skin*<br>More in oral<br>No difference<br>More in oral<br>More in oral<br>No difference<br>No difference<br>No difference	[71]<br>[70]<br>[73]<br>[61]<br>[61]<br>[61]<br>[61]<br>[61]<br>[61]<br>[71]
IL-1α	Primary human keratinocytes	TNF-α<br>TNF-α+IFN-γ	24 hours<br>24 hours	No difference<br>No difference	[61, 71]<br>[61]
IL-1β	Human tissue equivalent	UVB	24 hours	More in oral	[70]
TNF-α	Human tissue equivalent<br>Primary human keratinocytes	UVB<br>IL-1β	24 hours<br>2, 4, 12, 24, 48, 72 hours	More in oral<br>More in skin*	[70]<br>[73]
IL-10	Human tissue equivalent	UVB	24 hours	No difference	[70]
IL-18	Human tissue equivalent<br>Primary human keratinocytes	CA<br>TNF-α	24 hours<br>24 hours	More in skin<br>More in skin	[71]<br>[71]

*no direct comparison/statistics

Table S2: Direct comparison between skin and oral wound healing in processes involved in the proliferation phase

Proliferation phase	model			Difference	Ref
(3-7 days)	Species	stimulus	time		
Wound closure (macroscopic)	Human<br>Pig<br>Pig<br>Rat	3-5 mm biopsy<br>2x12 mm excision<br>15x17 mm excision<br>4 mm biopsy	9-15 days<br>14-49 days<br>28-60 days<br>1-7 days	Faster in oral<br>Faster in oral<br>Faster in oral<br>Faster in oral	[36]<br>[95]<br>[21]<br>[59]
Wound re-epithelialization (histology)	Pig<br>Pig<br>Human<br>Mouse	2x12 mm excision<br>15x17 mm excision<br>3-5 mm biopsy<br>1 mm biopsy	4 days<br>28 days<br>3-6 days<br>1-3 days	No difference<br>No difference<br>Faster in oral<br>Faster in oral	[95]<br>[21]<br>[36]<br>[69, 96]
Fibroblast migration	Primary human fibroblasts	Baseline	2 days<br>4 days	No difference<br>Faster in oral	[117]<br>[116]
Fibroblast proliferation	Primary human fibroblasts	Baseline<br><br><br><br><br>3D culture<br>TGF-β1	8 days<br>2 days<br>1-6 days<br>3-14 days<br>3-15 days<br>24 hours<br>24 hours	No difference<br>No difference<br>No difference<br>Faster in oral<br>Faster in oral<br>No difference<br>Increase in skin<br>Decrease in oral	[116]<br>[117]<br>[119]<br>[83]<br>[115]<br>[118]<br>[125]<br>[125]
Fibroblast contraction	Primary human fibroblasts	Baseline		More in oral	[103, 117, 127, 128]
α-SMA expression	Primary human fibroblasts	Baseline		More in skin<br>More in oral	[103, 127]<br>[117]
Fibroblast integrin expression	Primary human fibroblasts	Baseline		More in skin	[121]
Keratinocyte migration	Mouse/Human primary keratinocytes	Baseline		Faster in oral	[16]
Keratinocyte proliferation	Mouse/Human primary keratinocytes	Baseline		More in oral	[16, 115]
β6 Integrin	Pig	2x12 mm excision	0, 3, 7, 14, 21, 35, 49 days	More in oral	[123]

**Growth factors**	**model**			**Difference**	**Ref**
in vivo	**Species**	**stimulus**	**time**		
TGF-β	Pig	15x17 mm excision	60	More in skin	[21]
TGF-β1	Pig<br>Mice	2x12 mm excision<br>1 mm biopsy	0, 3, 7, 14, 21, 35, 49 days<br>12, 24, 48 hours<br>12, 24, 48, 72 hours	No difference<br>More in skin<br>More in skin	[123]<br>[96]<br>[69]
TGF-β3	Pig<br>Mice	2x12 mm excision<br>1 mm biopsy	0, 3, 7, 14, 21, 35, 49 days<br>12, 24, 48 hours	More in skin<br>No difference	[123]<br>[96]
pSMAD	Pig	2x12 mm excision	Baseline<br>60 days	More in oral<br>More in skin	[21]<br>[21]
FGF2/bFGF	Mice<br>Rat	1 mm biopsy<br>4 mm biopsy	1, 2, 3, 5, 7 days<br>0, 1, 2, 3, 4, 5 days	No difference<br>More in oral	[19]<br>[59]
VEGF	Mice<br>Rat	1 mm biopsy<br>4 mm biopsy	1, 2, 3, 5, 7 days<br>0, 1, 2 days<br>4, 5 days	More in skin/no difference<br>More in oral<br>More in skin	[19]<br>[59]<br>[59]
PDGF	Rat	4 mm biopsy	0, 1, 2, 3, 6, 7 days	More in oral	[59]
EGF	Rat	4 mm biopsy	1, 2, 3, 4, 5, 6 days	More in oral	[59]
HIF-1α	Mice	1 mm biopsy	6-24h	More in skin	[99]
**Growth factors**<br>***in vitro***					
TGF-β1	Primary human fibroblasts	3D culture	7 days	More in skin	[83]
TGF-β2	Primary human fibroblasts	3D culture	7 days	More in oral	[83]
TGF-β3	Primary human fibroblasts	3D culture	7 days	More in oral<br>More in skin	[96]<br>[83]
pSMAD	Primary human fibroblasts	3D culture	7 days	More in skin	[83]
FGF2	Primary human fibroblasts	3D culture	7 days	No difference	[83]
FGF7 (KGF)	Primary human fibroblasts	baseline	Baseline	More in oral	[103]
HGF	Primary human fibroblasts	baseline	Baseline	More in oral	[103]
KGF	Primary human fibroblasts	Baseline	Baseline	More in oral	[103]
VEGF-α	Primary human fibroblasts	3D culture	7 days	More in oral	[83]

Table S3: Direct comparison between skin and oral wound healing in processes involved in the remodeling phase

**Remodeling**	**model**			**difference**	**(ref)**
**(days-weeks-months)**	**species**	**stimulus**	**Time** (h)		
Scarring (macroscopically)	Pig	2x12 mm excision<br>15x27 mm excision	49 days<br>42, 46, 49 days<br>60 days	More in skin	[123]<br>[95]<br>[21]
Scarring (microscopically)	Pig	2x12 mm excision	49 days	More in skin	[95]
Contraction (macroscopically)	Pig	15x27 mm excision	60 days	More in skin	[21]
Epithelial thickness	Pig	15x27 mm excision	60 days	More in skin	[21]
Rete ridges	Pig	15x27 mm excision	60 days	More in oral	[21]
**Cells**					
Macrophages<br>(CD169)<br>(CD163)<br>(CD86)	 Pig<br>Pig<br>Human	 1 mm biopsy<br>15x27 mm excision<br>1 mm biopsy	 ?<br>60 days<br>1-3-6 days	 More in skin<br>More in skin?<br>More in skin?	 [69]<br>[21]<br>[36]
Mast cells	Pigs	15x27 mm excision	60 days	More in skin	[21]
Myofibroblasts (a-SMA)	Pig<br>Rat	15x27 mm excision<br>Collagen implant	60 days<br>1-2-4-8-16 weeks	More in oral<br>More in oral (1-2 weeks)	[21]<br>[130]
Fibroblasts	Pigs	15x27 mm excision	60 days	More in skin	[21]
Blood vessels	Pig<br>Rat	15x27 mm excision<br>Collagen implant	60 days<br>1-2-4-8-16 weeks	No difference/ More in skin<br>More in oral (1-2-4 weeks)	[21]<br>[130]
**Extracellular matrix *in vivo***					
Collagen density	Pig	15x27 mm excision	60 days	oral	[21]
Collagen maturity	Pig	15x27 mm excision	60 days	oral	[21]

Collagen fiber thickness	mice	1 mm biopsy	3-5-7-14 days	skin	[96]
Pro-collagen 1	Pig	2x12 mm excision	3-7-14-21-35-49 days	no difference/ skin	[95]
Fibronectin	Pig	2x12 mm excision	3-7-14-21-35-49 days	More in skin	[95]
**Extracellular matrix *in vitro***					
Collagen 1 (mRNA)<br>Collagen 1 (protein)	Human fibroblasts	3D culture<br><br>hypoxia	1 week<br>2 days<br>2 days	More in skin<br>More in oral<br>No difference	[83]<br>[113]<br>[113]
Collagen 3 (mRNA)<br>Collagen 3 (protein)	Human fibroblasts	3D culture<br><br>hypoxia	1 week<br>2 days<br>2 days	More in skin<br>More in skin<br>More in skin	[83]<br>[113]<br>[113]
Elastin-1 (mRNA)	Human fibroblasts	3D culture	1 week	More in skin	[83]
Fibronectin - EDA<br>- EDB	Human fibroblasts	3D culture	7 days	More in oral<br>No difference	[83]<br>[83]
Emilin-1/-2 (mRNA)	Human fibroblasts	3D culture	1 week	No difference	[83]
Fibrillin-1 (mRNA)	Human fibroblasts	3D culture	1 week	No difference	[83]
SPARC-1 (mRNA)	Human fibroblasts	3D culture	1 week	More in skin	[83]
Tenascin-C (mRNA)	Human fibroblasts	3D culture	1 week	More in skin	[83]
Total MMP expression (mRNA)	Human fibroblasts	3D culture	1 week	More in oral	[83]
MMP1 (active)<br><br><br>MMP1 (mRNA)	Human tissue equivalent<br>Human fibroblasts<br><br>Human fibroblasts	3D culture<br>3D culture<br><br>3D culture	1, 2, 3 weeks<br>1, 2, 3, 7, 14 days<br>1 week<br>1 week	No difference<br>No difference<br>No difference<br>More in oral	[136]<br>[136]<br>[83]<br>[83]

MMP2 (active)	Human fibroblasts	3D culture, baseline	1, 2, 3 days	No difference	[128]
			1 week	More in oral	[128]
MMP2 (protein)	Human tissue equivalents	3D culture, baseline	1, 2, 3 weeks	No difference	[134]
	Human fibroblasts	3D culture, baseline	1, 2, 3 days, 1-6 weeks	No difference	[134]
			1, 2, 3, 7 days	No difference	[128]
MMP2 (mRNA)	Human fibroblasts	3D culture, baseline	1 week	No difference	[83, 128]
MMP3 (active)	Human fibroblasts	3D culture, baseline	2 days	More in oral	[127]
MMP3 (protein)			2 days	More in oral	[113, 127]
		hypoxia	2 days	More in oral	[113]
MMP3 (mRNA)		3D culture, baseline	1 day	More in oral	[127]
			1 week	More in oral	[83]
MMP7 (mRNA)	Human fibroblasts	3D culture	1 week	More in skin	[83]
MMP9 (active)	Human tissue equivalents	Baseline	1-4 weeks	More in skin*	[134]
	Human fibroblasts	3D culture	1, 2, 3, 7, 14 days	More in oral*	[134]
MMP9 (protein)	Human fibroblasts	3D culture	1, 2, 3, 7 days	More in oral*	[134]
MMP10 (active)	Human fibroblasts	3D culture	1 week	More in oral	[83]
MMP10 (protein)		3D culture	2 days	More in oral	[113]
		Hypoxia	2 days	More in oral	[113]
MMP10 (mRNA)	Human fibroblasts	3D culture	1 week	More in oral	[83]
MMP11 (mRNA)	Human fibroblasts	3D culture	1 week	More in skin	[83]
MMP12 (mRNA)	Human fibroblasts	3D culture	1 week	No difference	[83]
TIMP1 (active)	Human fibroblasts	3D culture	1 week	More in skin	[128]
TIMP1(mRNA)			1 day	No difference	[128]
			1 week	No difference	[83]
TIMP2 (active)	Human fibroblasts	3D culture	1 week	More in skin	[128]
TIMP2 (mRNA)			1 day	No difference	[128]
			1 week	No difference	[83]
TIMP3 (mRNA)	Human fibroblasts	3D culture	1 week	No difference	[83]
TIMP4 (mRNA)	Human fibroblasts	3D culture	1 week	More in oral	[83]

References

1. Guo, S.; Dipietro, L. A. Factors affecting wound healing. *J Dent Res* **2010**, *89*, 219-229. doi:10.1177/0022034509359125
2. Chiang, R. S.; Borovikova, A. A.; King, K.; Banyard, D. A.; Lalezari, S.; Toranto, J. D.; Paydar, K. Z.; Wirth, G. A.; Evans, G. R.; Widgerow, A. D. Current concepts related to hypertrophic scarring in burn injuries. *Wound Repair Regen* **2016**, *24*, 466-477. doi:10.1111/wrr.12432
3. Finnerty, C. C.; Jeschke, M. G.; Branski, L. K.; Barret, J. P.; Dziewulski, P.; Herndon, D. N. Hypertrophic scarring: the greatest unmet challenge after burn injury. *Lancet* **2016**, *388*, 1427-1436. doi:10.1016/S0140-6736(16)31406-4
4. van den Broek, L. J.; van der Veer, W. M.; de Jong, E. H.; Gibbs, S.; Niessen, F. B. Suppressed inflammatory gene expression during human hypertrophic scar compared to normotrophic scar formation. *Exp Dermatol* **2015**, *24*, 623-629. doi:10.1111/exd.12739
5. van der Veer, W. M.; Niessen, F. B.; Ferreira, J. A.; Zwiers, P. J.; de Jong, E. H.; Middelkoop, E.; Molema, G. Time course of the angiogenic response during normotrophic and hypertrophic scar formation in humans. *Wound Repair Regen* **2011**, *19*, 292-301. doi:10.1111/j.1524-475X.2011.00692.x
6. Linehan, J. L.; Harrison, O. J.; Han, S. J.; Byrd, A. L.; Vujkovic-Cvijin, I.; Villarino, A. V.; Sen, S. K.; Shaik, J.; Smelkinson, M.; Tamoutounour, S.; Collins, N.; Bouladoux, N.; Dzutsev, A.; Rosshart, S. P.; Arbuckle, J. H.; Wang, C. R.; Kristie, T. M.; Rehermann, B.; Trinchieri, G.; Brenchley, J. M.; O'Shea, J. J.; Belkaid, Y. Non-classical Immunity Controls Microbiota Impact on Skin Immunity and Tissue Repair. *Cell* **2018**, *172*, 784-796 e718. doi:10.1016/j.cell.2017.12.033
7. Belkaid, Y.; Segre, J. A. Dialogue between skin microbiota and immunity. *Science* **2014**, *346*, 954-959. doi:10.1126/science.1260144
8. Lai, Y.; Di Nardo, A.; Nakatsuji, T.; Leichtle, A.; Yang, Y.; Cogen, A. L.; Wu, Z. R.; Hooper, L. V.; Schmidt, R. R.; von Aulock, S.; Radek, K. A.; Huang, C. M.; Ryan, A. F.; Gallo, R. L. Commensal bacteria regulate Toll-like receptor 3-dependent inflammation after skin injury. *Nat Med* **2009**, *15*, 1377-1382. doi:10.1038/nm.2062
9. Zheng, D.; Liwinski, T.; Elinav, E. Interaction between microbiota and immunity in health and disease. *Cell Res* **2020**, *30*, 492-506. doi:10.1038/s41422-020-0332-7
10. Kido, D.; Mizutani, K.; Takeda, K.; Mikami, R.; Matsuura, T.; Iwasaki, K.; Izumi, Y. Impact of diabetes on gingival wound healing via oxidative stress. *PLoS One* **2017**, *12*, e0189601. doi:10.1371/journal.pone.0189601
11. Evans, E. W. Treating Scars on the Oral Mucosa. *Facial Plast Surg Clin North Am* **2017**, *25*, 89-97. doi:10.1016/j.fsc.2016.08.008
12. Semlali, A.; Chakir, J.; Goulet, J. P.; Chmielewski, W.; Rouabhia, M. Whole cigarette smoke promotes human gingival epithelial cell apoptosis and inhibits cell repair processes. *J Periodontal Res* **2011**, *46*, 533-541. doi:10.1111/j.1600-0765.2011.01370.x
13. Presland, R. B.; Jurevic, R. J. Making sense of the epithelial barrier: what molecular biology and genetics tell us about the functions of oral mucosal and epidermal tissues. *J Dent Educ* **2002**, *66*, 564-574.
14. Qin, R.; Steel, A.; Fazel, N. Oral mucosa biology and salivary biomarkers. *Clin Dermatol* **2017**, *35*, 477-483. doi:10.1016/j.clindermatol.2017.06.005
15. Gibbs, S.; Ponec, M. Intrinsic regulation of differentiation markers in human epidermis, hard palate and buccal mucosa. *Arch Oral Biol* **2000**, *45*, 149-158. doi:10.1016/s0003-9969(99)00116-8
16. Turabelidze, A.; Guo, S.; Chung, A. Y.; Chen, L.; Dai, Y.; Marucha, P. T.; DiPietro, L. A. Intrinsic differences between oral and skin keratinocytes. *PLoS One* **2014**, *9*, e101480. doi:10.1371/journal.pone.0101480
17. Winning, T. A.; Townsend, G. C. Oral mucosal embryology and histology. *Clin Dermatol* **2000**, *18*, 499-511. doi:10.1016/s0738-081x(00)00140-1
18. Glim, J. E.; Beelen, R. H.; Niessen, F. B.; Everts, V.; Ulrich, M. M. The number of immune cells is lower in healthy oral mucosa compared to skin and does not increase after scarring. *Arch Oral Biol* **2015**, *60*, 272-281. doi:10.1016/j.archoralbio.2014.10.008
19. Szpaderska, A. M.; Walsh, C. G.; Steinberg, M. J.; DiPietro, L. A. Distinct patterns of angiogenesis in oral and skin wounds. *J Dent Res* **2005**, *84*, 309-314. doi:10.1177/154405910508400403
20. Glim, J. E.; Everts, V.; Niessen, F. B.; Ulrich, M. M.; Beelen, R. H. Extracellular matrix components of oral mucosa differ from skin and resemble that of foetal skin. *Arch Oral Biol* **2014**, *59*, 1048-1055. doi:10.1016/j.archoralbio.2014.05.019
21. Mak, K.; Manji, A.; Gallant-Behm, C.; Wiebe, C.; Hart, D. A.; Larjava, H.; Hakkinen, L. Scarless healing of oral mucosa is characterized by faster resolution of inflammation and control of myofibroblast action compared to skin wounds in the red Duroc pig model. *J Dermatol Sci* **2009**, *56*, 168-180. doi:10.1016/j.jdermsci.2009.09.005

22. Hsieh, P. C.; Jin, Y. T.; Chang, C. W.; Huang, C. C.; Liao, S. C.; Yuan, K. Elastin in oral connective tissue modulates the keratinization of overlying epithelium. *J Clin Periodontol* **2010**, *37*, 705-711. doi:10.1111/j.1600-051X.2010.01542.x
23. Dyson, M.; Young, S. R.; Hart, J.; Lynch, J. A.; Lang, S. Comparison of the effects of moist and dry conditions on the process of angiogenesis during dermal repair. *J Invest Dermatol* **1992**, *99*, 729-733. doi:10.1111/1523-1747.ep12614460
24. Junker, J. P.; Kamel, R. A.; Caterson, E. J.; Eriksson, E. Clinical Impact Upon Wound Healing and Inflammation in Moist, Wet, and Dry Environments. *Adv Wound Care (New Rochelle)* **2013**, *2*, 348-356. doi:10.1089/wound.2012.0412
25. Svensjo, T.; Pomahac, B.; Yao, F.; Slama, J.; Eriksson, E. Accelerated healing of full-thickness skin wounds in a wet environment. *Plast Reconstr Surg* **2000**, *106*, 602-612; discussion 613-604.
26. Vogt, P. M.; Andree, C.; Breuing, K.; Liu, P. Y.; Slama, J.; Helo, G.; Eriksson, E. Dry, moist, and wet skin wound repair. *Ann Plast Surg* **1995**, *34*, 493-499; discussion 499-500. doi:10.1097/00000637-199505000-00007
27. Brand, H. S.; Ligtenberg, A. J.; Veerman, E. C. Saliva and wound healing. *Monogr Oral Sci* **2014**, *24*, 52-60. doi:10.1159/000358784
28. Lei, X.; Cheng, L.; Lin, H.; Pang, M.; Yao, Z.; Chen, C.; Forouzanfar, T.; Bikker, F. J.; Wu, G.; Cheng, B. Human Salivary Histatin-1 Is More Efficacious in Promoting Acute Skin Wound Healing Than Acellular Dermal Matrix Paste. *Front Bioeng Biotechnol* **2020**, *8*, 999. doi:10.3389/fbioe.2020.00999
29. Ma, D.; Sun, W.; Nazmi, K.; Veerman, E. C. I.; Bikker, F. J.; Jaspers, R. T.; Bolscher, J. G. M.; Wu, G. Salivary Histatin 1 and 2 Are Targeted to Mitochondria and Endoplasmic Reticulum in Human Cells. *Cells* **2020**, *9*. doi:10.3390/cells9040795
30. Bodner, L.; Knyszynski, A.; Adler-Kunin, S.; Danon, D. The effect of selective desalivation on wound healing in mice. *Exp Gerontol* **1991**, *26*, 357-363. doi:10.1016/0531-5565(91)90047-p
31. Bodner, L.; Dayan, D.; Pinto, Y.; Hammel, I. Characteristics of palatal wound healing in desalivated rats. *Arch Oral Biol* **1993**, *38*, 17-21. doi:10.1016/0003-9969(93)90149-g
32. Bodner, L.; Kaffe, I.; Cohen, Z.; Dayan, D. Long-term effect of desalivation on extraction wound healing: a densitometric study in rats. *Dentomaxillofac Radiol* **1993**, *22*, 195-198. doi:10.1259/dmfr.22.4.8181646
33. Caselli, E.; Fabbri, C.; D'Accolti, M.; Soffritti, I.; Bassi, C.; Mazzacane, S.; Franchi, M. Defining the oral microbiome by whole-genome sequencing and resistome analysis: the complexity of the healthy picture. *BMC Microbiol* **2020**, *20*, 120. doi:10.1186/s12866-020-01801-y
34. Deo, P. N.; Deshmukh, R. Oral microbiome: Unveiling the fundamentals. *J Oral Maxillofac Pathol* **2019**, *23*, 122-128. doi:10.4103/jomfp.JOMFP_304_18
35. Human Microbiome Project, C. Structure, function and diversity of the healthy human microbiome. *Nature* **2012**, *486*, 207-214. doi:10.1038/nature11234
36. Iglesias-Bartolome, R.; Uchiyama, A.; Molinolo, A. A.; Abusleme, L.; Brooks, S. R.; Callejas-Valera, J. L.; Edwards, D.; Doci, C.; Asselin-Labat, M. L.; Onaitis, M. W.; Moutsopoulos, N. M.; Gutkind, J. S.; Morasso, M. I. Transcriptional signature primes human oral mucosa for rapid wound healing. *Sci Transl Med* **2018**, *10*. doi:10.1126/scitranslmed.aap8798
37. Verma, D.; Garg, P. K.; Dubey, A. K. Insights into the human oral microbiome. *Arch Microbiol* **2018**, *200*, 525-540. doi:10.1007/s00203-018-1505-3
38. Krishnan, K.; Chen, T.; Paster, B. J. A practical guide to the oral microbiome and its relation to health and disease. *Oral Dis* **2017**, *23*, 276-286. doi:10.1111/odi.12509
39. Dewhirst, F. E.; Chen, T.; Izard, J.; Paster, B. J.; Tanner, A. C.; Yu, W. H.; Lakshmanan, A.; Wade, W. G. The human oral microbiome. *J Bacteriol* **2010**, *192*, 5002-5017. doi:10.1128/JB.00542-10
40. Grice, E. A.; Kong, H. H.; Conlan, S.; Deming, C. B.; Davis, J.; Young, A. C.; Program, N. C. S.; Bouffard, G. G.; Blakesley, R. W.; Murray, P. R.; Green, E. D.; Turner, M. L.; Segre, J. A. Topographical and temporal diversity of the human skin microbiome. *Science* **2009**, *324*, 1190-1192. doi:10.1126/science.1171700
41. Shang, L.; Deng, D.; Buskermolen, J. K.; Janus, M. M.; Krom, B. P.; Roffel, S.; Waaijman, T.; van Loveren, C.; Crielaard, W.; Gibbs, S. Multi-species oral biofilm promotes reconstructed human gingiva epithelial barrier function. *Sci Rep* **2018**, *8*, 16061. doi:10.1038/s41598-018-34390-y
42. Laheij, A. M.; de Soet, J. J.; Veerman, E. C.; Bolscher, J. G.; van Loveren, C. The influence of oral bacteria on epithelial cell migration in vitro. *Mediators Inflamm* **2013**, *2013*, 154532. doi:10.1155/2013/154532
43. Karin, M.; Clevers, H. Reparative inflammation takes charge of tissue regeneration. *Nature* **2016**, *529*, 307-315. doi:10.1038/nature17039
44. Church, D.; Elsayed, S.; Reid, O.; Winston, B.; Lindsay, R. Burn wound infections. *Clin Microbiol Rev* **2006**, *19*, 403-434. doi:10.1128/CMR.19.2.403-434.2006

45. van 't Hof, W.; Veerman, E. C.; Nieuw Amerongen, A. V.; Ligtenberg, A. J. Antimicrobial defense systems in saliva. *Monogr Oral Sci* **2014**, *24*, 40-51. doi:10.1159/000358783
46. Amerongen, A. V.; Veerman, E. C. Saliva--the defender of the oral cavity. *Oral Dis* **2002**, *8*, 12-22. doi:10.1034/j.1601-0825.2002.1o816.x
47. Bikker, F. J.; End, C.; Ligtenberg, A. J. M.; Blaich, S.; Lyer, S.; Renner, M.; Wittig, R.; Nazmi, K.; van Nieuw Amerongen, A.; Poustka, A.; Veerman, E. C. I.; Mollenhauer, J. The scavenging capacity of DMBT1 is impaired by germline deletions. *Immunogenetics* **2017**, *69*, 401-407. doi:10.1007/s00251-017-0982-x
48. Groenink, J.; Ligtenberg, A. J.; Veerman, E. C.; Bolscher, J. G.; Nieuw Amerongen, A. V. Interaction of the salivary low-molecular-weight mucin (MG2) with Actinobacillus actinomycetemcomitans. *Antonie Van Leeuwenhoek* **1996**, *70*, 79-87. doi:10.1007/BF00393572
49. Ligtenberg, T. J.; Bikker, F. J.; Groenink, J.; Tornoe, I.; Leth-Larsen, R.; Veerman, E. C.; Nieuw Amerongen, A. V.; Holmskov, U. Human salivary agglutinin binds to lung surfactant protein-D and is identical with scavenger receptor protein gp-340. *Biochem J* **2001**, *359*, 243-248. doi:10.1042/0264-6021:3590243
50. Murray, P. A.; Prakobphol, A.; Lee, T.; Hoover, C. I.; Fisher, S. J. Adherence of oral streptococci to salivary glycoproteins. *Infect Immun* **1992**, *60*, 31-38. doi:10.1128/IAI.60.1.31-38.1992
51. Marsh, P. D.; Do, T.; Beighton, D.; Devine, D. A. Influence of saliva on the oral microbiota. *Periodontol 2000* **2016**, *70*, 80-92. doi:10.1111/prd.12098
52. Molhoek, E. M.; den Hertog, A. L.; de Vries, A. M.; Nazmi, K.; Veerman, E. C.; Hartgers, F. C.; Yazdanbakhsh, M.; Bikker, F. J.; van der Kleij, D. Structure-function relationship of the human antimicrobial peptide LL-37 and LL-37 fragments in the modulation of TLR responses. *Biol Chem* **2009**, *390*, 295-303. doi:10.1515/BC.2009.037
53. Krom, B. P.; Oskam, J. Microbial biofilms and wound healing: an ecological hypothesis. *Phlebology* **2014**, *29*, 168-173. doi:10.1177/0268355514528845
54. Abusleme, L.; Hoare, A.; Hong, B. Y.; Diaz, P. I. Microbial signatures of health, gingivitis, and periodontitis. *Periodontol 2000* **2021**, *86*, 57-78. doi:10.1111/prd.12362
55. Glazko, A. J.; Greenberg, D.M. The mechanism of the action of saliva in blood coagulation. *Am J Physiol* **1938**, *125*, 108-112.
56. Berckmans, R. J.; Sturk, A.; van Tienen, L. M.; Schaap, M. C.; Nieuwland, R. Cell-derived vesicles exposing coagulant tissue factor in saliva. *Blood* **2011**, *117*, 3172-3180. doi:10.1182/blood-2010-06-290460
57. Ridiandries, A.; Tan, J. T. M.; Bursill, C. A. The Role of Chemokines in Wound Healing. *Int J Mol Sci* **2018**, *19*. doi:10.3390/ijms19103217
58. Eisinger, F.; Patzelt, J.; Langer, H. F. The Platelet Response to Tissue Injury. *Front Med (Lausanne)* **2018**, *5*, 317. doi:10.3389/fmed.2018.00317
59. Kong, X.; Fu, J.; Shao, K.; Wang, L.; Lan, X.; Shi, J. Biomimetic hydrogel for rapid and scar-free healing of skin wounds inspired by the healing process of oral mucosa. *Acta Biomater* **2019**, *100*, 255-269. doi:10.1016/j.actbio.2019.10.011
60. Wilgus, T. A. Inflammation as an orchestrator of cutaneous scar formation: a review of the literature. *Plast Aesthet Res* **2020**, *7*. doi:10.20517/2347-9264.2020.150
61. Li, J.; Farthing, P. M.; Ireland, G. W.; Thornhill, M. H. IL-1 alpha and IL-6 production by oral and skin keratinocytes: similarities and differences in response to cytokine treatment in vitro. *J Oral Pathol Med* **1996**, *25*, 157-162. doi:10.1111/j.1600-0714.1996.tb00213.x
62. Butzelaar, L.; Schooneman, D. P.; Soykan, E. A.; Talhout, W.; Ulrich, M. M.; van den Broek, L. J.; Gibbs, S.; Beelen, R. H.; Mink van der Molen, A. B.; Niessen, F. B. Inhibited early immunologic response is associated with hypertrophic scarring. *Exp Dermatol* **2016**, *25*, 797-804. doi:10.1111/exd.13100
63. de Bakker, E.; van der Putten, M. A. M.; Heymans, M. W.; Spiekstra, S. W.; Waaijman, T.; Butzelaar, L.; Negenborn, V. L.; Beekman, V. K.; Akpinar, E. O.; Rustemeyer, T.; Niessen, F. B.; Gibbs, S. Prognostic tools for hypertrophic scar formation based on fundamental differences in systemic immunity. *Exp Dermatol* **2021**, *30*, 169-178. doi:10.1111/exd.14139
64. Bootun, R. Effects of immunosuppressive therapy on wound healing. *Int Wound J* **2013**, *10*, 98-104. doi:10.1111/j.1742-481X.2012.00950.x
65. Larouche, J.; Sheoran, S.; Maruyama, K.; Martino, M. M. Immune Regulation of Skin Wound Healing: Mechanisms and Novel Therapeutic Targets. *Adv Wound Care (New Rochelle)* **2018**, *7*, 209-231. doi:10.1089/wound.2017.0761
66. Sanford, J. A.; Zhang, L. J.; Williams, M. R.; Gangoiti, J. A.; Huang, C. M.; Gallo, R. L. Inhibition of HDAC8 and HDAC9 by microbial short-chain fatty acids breaks immune tolerance of the epidermis to TLR ligands. *Sci Immunol* **2016**, *1*. doi:10.1126/sciimmunol.aah4609

67. Sciubba, J. J.; Waterhouse, J. P.; Meyer, J. A fine structural comparison of the healing of incisional wounds of mucosa and skin. *J Oral Pathol* **1978**, *7*, 214-227. doi:10.1111/j.1600-0714.1978.tb01596.x
68. Phillipson, M.; Kubes, P. The Healing Power of Neutrophils. *Trends Immunol* **2019**, *40*, 635-647. doi:10.1016/j.it.2019.05.001
69. Szpaderska, A. M.; Zuckerman, J. D.; DiPietro, L. A. Differential injury responses in oral mucosal and cutaneous wounds. *J Dent Res* **2003**, *82*, 621-626. doi:10.1177/154405910308200810
70. Breger, J.; Baeva, L.; Agrawal, A.; Shindell, E.; Godar, D. E. UVB-induced inflammatory cytokine release, DNA damage and apoptosis of human oral compared with skin tissue equivalents. *Photochem Photobiol* **2013**, *89*, 665-670. doi:10.1111/php.12030
71. Kosten, I. J.; Buskermolen, J. K.; Spiekstra, S. W.; de Gruijl, T. D.; Gibbs, S. Gingiva Equivalents Secrete Negligible Amounts of Key Chemokines Involved in Langerhans Cell Migration Compared to Skin Equivalents. *J Immunol Res* **2015**, *2015*, 627125. doi:10.1155/2015/627125
72. Li, J.; Ireland, G. W.; Farthing, P. M.; Thornhill, M. H. Epidermal and oral keratinocytes are induced to produce RANTES and IL-8 by cytokine stimulation. *J Invest Dermatol* **1996**, *106*, 661-666. doi:10.1111/1523-1747.ep12345482
73. Chen, L.; Arbieva, Z. H.; Guo, S.; Marucha, P. T.; Mustoe, T. A.; DiPietro, L. A. Positional differences in the wound transcriptome of skin and oral mucosa. *BMC Genomics* **2010**, *11*, 471. doi:10.1186/1471-2164-11-471
74. Li, J.; Mahiouz, D. L.; Farthing, P. M.; Haskard, D. O.; Thornhill, M. H. Heterogeneity of ICAM-1 expression, and cytokine regulation of ICAM-1 expression, in skin and oral keratinocytes. *J Oral Pathol Med* **1996**, *25*, 112-118. doi:10.1111/j.1600-0714.1996.tb00204.x
75. Tecchio, C.; Cassatella, M. A. Neutrophil-derived chemokines on the road to immunity. *Semin Immunol* **2016**, *28*, 119-128. doi:10.1016/j.smim.2016.04.003
76. Li, J.; Farthing, P. M.; Thornhill, M. H. Oral and skin keratinocytes are stimulated to secrete monocyte chemoattractant protein-1 by tumour necrosis factor-alpha and interferon-gamma. *J Oral Pathol Med* **2000**, *29*, 438-444. doi:10.1034/j.1600-0714.2000.290904.x
77. Boothby, I. C.; Cohen, J. N.; Rosenblum, M. D. Regulatory T cells in skin injury: At the crossroads of tolerance and tissue repair. *Sci Immunol* **2020**, *5*. doi:10.1126/sciimmunol.aaz9631
78. Landen, N. X.; Li, D.; Stahle, M. Transition from inflammation to proliferation: a critical step during wound healing. *Cell Mol Life Sci* **2016**, *73*, 3861-3885. doi:10.1007/s00018-016-2268-0
79. MacLeod, A. S.; Mansbridge, J. N. The Innate Immune System in Acute and Chronic Wounds. *Adv Wound Care (New Rochelle)* **2016**, *5*, 65-78. doi:10.1089/wound.2014.0608
80. Strbo, N.; Yin, N.; Stojadinovic, O. Innate and Adaptive Immune Responses in Wound Epithelialization. *Adv Wound Care (New Rochelle)* **2014**, *3*, 492-501. doi:10.1089/wound.2012.0435
81. Strazza, M.; Mor, A. Consider the chemokines: a review of the interplay between chemokines and T cell subset function. *Discov Med* **2017**, *24*, 31-39.
82. Viola, A.; Contento, R. L.; Molon, B. T cells and their partners: The chemokine dating agency. *Trends Immunol* **2006**, *27*, 421-427. doi:10.1016/j.it.2006.07.004
83. Mah, W.; Jiang, G.; Olver, D.; Cheung, G.; Kim, B.; Larjava, H.; Hakkinen, L. Human gingival fibroblasts display a non-fibrotic phenotype distinct from skin fibroblasts in three-dimensional cultures. *PLoS One* **2014**, *9*, e90715. doi:10.1371/journal.pone.0090715
84. Naik, S.; Bouladoux, N.; Wilhelm, C.; Molloy, M. J.; Salcedo, R.; Kastenmuller, W.; Deming, C.; Quinones, M.; Koo, L.; Conlan, S.; Spencer, S.; Hall, J. A.; Dzutsev, A.; Kong, H.; Campbell, D. J.; Trinchieri, G.; Segre, J. A.; Belkaid, Y. Compartmentalized control of skin immunity by resident commensals. *Science* **2012**, *337*, 1115-1119. doi:10.1126/science.1225152
85. Naik, S.; Bouladoux, N.; Linehan, J. L.; Han, S. J.; Harrison, O. J.; Wilhelm, C.; Conlan, S.; Himmelfarb, S.; Byrd, A. L.; Deming, C.; Quinones, M.; Brenchley, J. M.; Kong, H. H.; Tussiwand, R.; Murphy, K. M.; Merad, M.; Segre, J. A.; Belkaid, Y. Commensal-dendritic-cell interaction specifies a unique protective skin immune signature. *Nature* **2015**, *520*, 104-108. doi:10.1038/nature14052
86. Ng, M. F. The role of mast cells in wound healing. *Int Wound J* **2010**, *7*, 55-61. doi:10.1111/j.1742-481X.2009.00651.x
87. Do, D. V.; Ong, C. T.; Khoo, Y. T.; Carbone, A.; Lim, C. P.; Wang, S.; Mukhopadhyay, A.; Cao, X.; Cho, D. H.; Wei, X. Q.; Bellone, G.; Lim, I.; Phan, T. T. Interleukin-18 system plays an important role in keloid pathogenesis via epithelial-mesenchymal interactions. *Br J Dermatol* **2012**, *166*, 1275-1288. doi:10.1111/j.1365-2133.2011.10721.x
88. Lee, J.; Rodero, M. P.; Patel, J.; Moi, D.; Mazzieri, R.; Khosrotehrani, K. Interleukin-23 regulates interleukin-17 expression in wounds, and its inhibition accelerates diabetic wound healing through the alteration of macrophage polarization. *FASEB J* **2018**, *32*, 2086-2094. doi:10.1096/fj.201700773R

89. Matias, M. A.; Saunus, J. M.; Ivanovski, S.; Walsh, L. J.; Farah, C. S. Accelerated wound healing phenotype in Interleukin 12/23 deficient mice. *J Inflamm (Lond)* **2011**, *8*, 39. doi:10.1186/1476-9255-8-39
90. Nakayama, W.; Jinnin, M.; Tomizawa, Y.; Nakamura, K.; Kudo, H.; Inoue, K.; Makino, K.; Honda, N.; Kajihara, I.; Fukushima, S.; Ihn, H. Dysregulated interleukin-23 signalling contributes to the increased collagen production in scleroderma fibroblasts via balancing microRNA expression. *Rheumatology (Oxford)* **2017**, *56*, 145-155. doi:10.1093/rheumatology/kew336
91. Poindexter, N. J.; Williams, R. R.; Powis, G.; Jen, E.; Caudle, A. S.; Chada, S.; Grimm, E. A. IL-24 is expressed during wound repair and inhibits TGFalpha-induced migration and proliferation of keratinocytes. *Exp Dermatol* **2010**, *19*, 714-722. doi:10.1111/j.1600-0625.2010.01077.x
92. Rao, L. Z.; Wang, Y.; Zhang, L.; Wu, G.; Zhang, L.; Wang, F. X.; Chen, L. M.; Sun, F.; Jia, S.; Zhang, S.; Yu, Q.; Wei, J. H.; Lei, H. R.; Yuan, T.; Li, J.; Huang, X.; Cheng, B.; Zhao, J.; Xu, Y.; Mo, B. W.; Wang, C. Y.; Zhang, H. IL-24 deficiency protects mice against bleomycin-induced pulmonary fibrosis by repressing IL-4-induced M2 program in macrophages. *Cell Death Differ* **2021**, *28*, 1270-1283. doi:10.1038/s41418-020-00650-6
93. Stout, A. J.; Gresser, I.; Thompson, W. D. Inhibition of wound healing in mice by local interferon alpha/beta injection. *Int J Exp Pathol* **1993**, *74*, 79-85.
94. Pastar, I.; Stojadinovic, O.; Yin, N. C.; Ramirez, H.; Nusbaum, A. G.; Sawaya, A.; Patel, S. B.; Khalid, L.; Isseroff, R. R.; Tomic-Canic, M. Epithelialization in Wound Healing: A Comprehensive Review. *Adv Wound Care (New Rochelle)* **2014**, *3*, 445-464. doi:10.1089/wound.2013.0473
95. Wong, J. W.; Gallant-Behm, C.; Wiebe, C.; Mak, K.; Hart, D. A.; Larjava, H.; Hakkinen, L. Wound healing in oral mucosa results in reduced scar formation as compared with skin: evidence from the red Duroc pig model and humans. *Wound Repair Regen* **2009**, *17*, 717-729. doi:10.1111/j.1524-475X.2009.00531.x
96. Schrementi, M. E.; Ferreira, A. M.; Zender, C.; DiPietro, L. A. Site-specific production of TGF-beta in oral mucosal and cutaneous wounds. *Wound Repair Regen* **2008**, *16*, 80-86. doi:10.1111/j.1524-475X.2007.00320.x
97. DiPietro, L. A. Angiogenesis and wound repair: when enough is enough. *J Leukoc Biol* **2016**, *100*, 979-984. doi:10.1189/jlb.4MR0316-102R
98. Khan, A. Detection and quantitation of forty eight cytokines, chemokines, growth factors and nine acute phase proteins in healthy human plasma, saliva and urine. *J Proteomics* **2012**, *75*, 4802-4819. doi:10.1016/j.jprot.2012.05.018
99. Chen, L.; Gajendrareddy, P. K.; DiPietro, L. A. Differential expression of HIF-1alpha in skin and mucosal wounds. *J Dent Res* **2012**, *91*, 871-876. doi:10.1177/0022034512454435
100. Wietecha, M. S.; Cerny, W. L.; DiPietro, L. A. Mechanisms of vessel regression: toward an understanding of the resolution of angiogenesis. *Curr Top Microbiol Immunol* **2013**, *367*, 3-32. doi:10.1007/82_2012_287
101. Xue, M.; Jackson, C. J. Extracellular Matrix Reorganization During Wound Healing and Its Impact on Abnormal Scarring. *Adv Wound Care (New Rochelle)* **2015**, *4*, 119-136. doi:10.1089/wound.2013.0485
102. Seeger, M. A.; Paller, A. S. The Roles of Growth Factors in Keratinocyte Migration. *Adv Wound Care (New Rochelle)* **2015**, *4*, 213-224. doi:10.1089/wound.2014.0540
103. Shannon, D. B.; McKeown, S. T.; Lundy, F. T.; Irwin, C. R. Phenotypic differences between oral and skin fibroblasts in wound contraction and growth factor expression. *Wound Repair Regen* **2006**, *14*, 172-178. doi:10.1111/j.1743-6109.2006.00107.x
104. Jahovic, N.; Guzel, E.; Arbak, S.; Yegen, B. C. The healing-promoting effect of saliva on skin burn is mediated by epidermal growth factor (EGF): role of the neutrophils. *Burns* **2004**, *30*, 531-538. doi:10.1016/j.burns.2004.02.007
105. Oudhoff, M. J.; Bolscher, J. G.; Nazmi, K.; Kalay, H.; van 't Hof, W.; Amerongen, A. V.; Veerman, E. C. Histatins are the major wound-closure stimulating factors in human saliva as identified in a cell culture assay. *FASEB J* **2008**, *22*, 3805-3812. doi:10.1096/fj.08-112003
106. Zelles, T.; Purushotham, K. R.; Macauley, S. P.; Oxford, G. E.; Humphreys-Beher, M. G. Saliva and growth factors: the fountain of youth resides in us all. *J Dent Res* **1995**, *74*, 1826-1832. doi:10.1177/00220345950740120301
107. Rodrigues Neves, C.; Buskermolen, J.; Roffel, S.; Waaijman, T.; Thon, M.; Veerman, E.; Gibbs, S. Human saliva stimulates skin and oral wound healing in vitro. *J Tissue Eng Regen Med* **2019**, *13*, 1079-1092. doi:10.1002/term.2865
108. Oudhoff, M. J.; Kroeze, K. L.; Nazmi, K.; van den Keijbus, P. A.; van 't Hof, W.; Fernandez-Borja, M.; Hordijk, P. L.; Gibbs, S.; Bolscher, J. G.; Veerman, E. C. Structure-activity analysis of histatin, a potent wound healing peptide from human saliva: cyclization of histatin potentiates molar activity 1,000-fold. *FASEB J* **2009**, *23*, 3928-3935. doi:10.1096/fj.09-137588

109. Oudhoff, M. J.; van den Keijbus, P. A.; Kroeze, K. L.; Nazmi, K.; Gibbs, S.; Bolscher, J. G.; Veerman, E. C. Histatins enhance wound closure with oral and non-oral cells. *J Dent Res* **2009**, *88*, 846-850. doi:10.1177/0022034509342951
110. Haverman, T. M.; Laheij, A.; de Soet, J. J.; de Lange, J.; Rozema, F. R. Candida and Porphyromonas gingivalis: the effect on wound closure in vitro. *J Oral Microbiol* **2017**, *9*, 1328266. doi:10.1080/20002297.2017.1328266
111. De Ryck, T.; Vanlancker, E.; Grootaert, C.; Roman, B. I.; De Coen, L. M.; Vandenberghe, I.; Stevens, C. V.; Bracke, M.; Van de Wiele, T.; Vanhoecke, B. Microbial inhibition of oral epithelial wound recovery: potential role for quorum sensing molecules? *AMB Express* **2015**, *5*, 27. doi:10.1186/s13568-015-0116-5
112. Maquart, F. X.; Monboisse, J. C. Extracellular matrix and wound healing. *Pathol Biol (Paris)* **2014**, *62*, 91-95. doi:10.1016/j.patbio.2014.02.007
113. Hara-Saito, Y.; Kato, H.; Saito, N.; Shiomi, A.; Uenoyama, A.; Takagi, R.; Izumi, K. Distinct differences in hypoxic responses between human oral mucosa and skin fibroblasts in a 3D collagen matrix. *In Vitro Cell Dev Biol Anim* **2020**, *56*, 452-479. doi:10.1007/s11626-020-00458-1
114. Rousselle, P.; Montmasson, M.; Garnier, C. Extracellular matrix contribution to skin wound re-epithelialization. *Matrix Biol* **2019**, *75-76*, 12-26. doi:10.1016/j.matbio.2018.01.002
115. Roh, J. L.; Lee, J.; Kim, E. H.; Shin, D. Plasticity of oral mucosal cell sheets for accelerated and scarless skin wound healing. *Oral Oncol* **2017**, *75*, 81-88. doi:10.1016/j.oraloncology.2017.10.024
116. Boink, M. A.; van den Broek, L. J.; Roffel, S.; Nazmi, K.; Bolscher, J. G.; Gefen, A.; Veerman, E. C.; Gibbs, S. Different wound healing properties of dermis, adipose, and gingiva mesenchymal stromal cells. *Wound Repair Regen* **2016**, *24*, 100-109. doi:10.1111/wrr.12380
117. Lygoe, K. A.; Wall, I.; Stephens, P.; Lewis, M. P. Role of vitronectin and fibronectin receptors in oral mucosal and dermal myofibroblast differentiation. *Biol Cell* **2007**, *99*, 601-614. doi:10.1042/BC20070008
118. Stephens, P.; Davies, K. J.; al-Khateeb, T.; Shepherd, J. P.; Thomas, D. W. A comparison of the ability of intra-oral and extra-oral fibroblasts to stimulate extracellular matrix reorganization in a model of wound contraction. *J Dent Res* **1996**, *75*, 1358-1364. doi:10.1177/00220345960750060601
119. van Wyk, C. W.; Olivier, A.; Hoal-van Helden, E. G.; Grobler-Rabie, A. F. Growth of oral and skin fibroblasts from patients with oral submucous fibrosis. *J Oral Pathol Med* **1995**, *24*, 349-353. doi:10.1111/j.1600-0714.1995.tb01198.x
120. Palaiologou, A. A.; Yukna, R. A.; Moses, R.; Lallier, T. E. Gingival, dermal, and periodontal ligament fibroblasts express different extracellular matrix receptors. *J Periodontol* **2001**, *72*, 798-807. doi:10.1902/jop.2001.72.6.798
121. Guo, F.; Carter, D. E.; Mukhopadhyay, A.; Leask, A. Gingival fibroblasts display reduced adhesion and spreading on extracellular matrix: a possible basis for scarless tissue repair? *PLoS One* **2011**, *6*, e27097. doi:10.1371/journal.pone.0027097
122. Penn, J. W.; Grobbelaar, A. O.; Rolfe, K. J. The role of the TGF-beta family in wound healing, burns and scarring: a review. *Int J Burns Trauma* **2012**, *2*, 18-28.
123. Eslami, A.; Gallant-Behm, C. L.; Hart, D. A.; Wiebe, C.; Honardoust, D.; Gardner, H.; Hakkinen, L.; Larjava, H. S. Expression of integrin alphavbeta6 and TGF-beta in scarless vs scar-forming wound healing. *J Histochem Cytochem* **2009**, *57*, 543-557. doi:10.1369/jhc.2009.952572
124. Lee, H. G.; Eun, H. C. Differences between fibroblasts cultured from oral mucosa and normal skin: implication to wound healing. *J Dermatol Sci* **1999**, *21*, 176-182. doi:10.1016/s0923-1811(99)00037-7
125. Meran, S.; Luo, D. D.; Simpson, R.; Martin, J.; Wells, A.; Steadman, R.; Phillips, A. O. Hyaluronan facilitates transforming growth factor-beta1-dependent proliferation via CD44 and epidermal growth factor receptor interaction. *J Biol Chem* **2011**, *286*, 17618-17630. doi:10.1074/jbc.M111.226563
126. Aplin, A. C.; Zhu, W. H.; Fogel, E.; Nicosia, R. F. Vascular regression and survival are differentially regulated by MT1-MMP and TIMPs in the aortic ring model of angiogenesis. *Am J Physiol Cell Physiol* **2009**, *297*, C471-480. doi:10.1152/ajpcell.00019.2009
127. McKeown, S. T.; Barnes, J. J.; Hyland, P. L.; Lundy, F. T.; Fray, M. J.; Irwin, C. R. Matrix metalloproteinase-3 differences in oral and skin fibroblasts. *J Dent Res* **2007**, *86*, 457-462. doi:10.1177/154405910708600513
128. Stephens, P.; Davies, K. J.; Occleston, N.; Pleass, R. D.; Kon, C.; Daniels, J.; Khaw, P. T.; Thomas, D. W. Skin and oral fibroblasts exhibit phenotypic differences in extracellular matrix reorganization and matrix metalloproteinase activity. *Br J Dermatol* **2001**, *144*, 229-237. doi:10.1046/j.1365-2133.2001.04006.x
129. Enoch, S.; Peake, M. A.; Wall, I.; Davies, L.; Farrier, J.; Giles, P.; Kipling, D.; Price, P.; Moseley, R.; Thomas, D.; Stephens, P. 'Young' oral fibroblasts are geno/phenotypically distinct. *J Dent Res* **2010**, *89*, 1407-1413. doi:10.1177/0022034510377796

130. Jansen, R. G.; van Kuppevelt, T. H.; Daamen, W. F.; Kuijpers-Jagtman, A. M.; Von den Hoff, J. W. Tissue reactions to collagen scaffolds in the oral mucosa and skin of rats: environmental and mechanical factors. *Arch Oral Biol* **2008**, *53*, 376-387. doi:10.1016/j.archoralbio.2007.11.003
131. Walton, K. L.; Johnson, K. E.; Harrison, C. A. Targeting TGF-beta Mediated SMAD Signaling for the Prevention of Fibrosis. *Front Pharmacol* **2017**, *8*, 461. doi:10.3389/fphar.2017.00461
132. Xue, M.; Le, N. T.; Jackson, C. J. Targeting matrix metalloproteases to improve cutaneous wound healing. *Expert Opin Ther Targets* **2006**, *10*, 143-155. doi:10.1517/14728222.10.1.143
133. Van den Steen, P. E.; Proost, P.; Wuyts, A.; Van Damme, J.; Opdenakker, G. Neutrophil gelatinase B potentiates interleukin-8 tenfold by aminoterminal processing, whereas it degrades CTAP-III, PF-4, and GRO-alpha and leaves RANTES and MCP-2 intact. *Blood* **2000**, *96*, 2673-2681.
134. McQuibban, G. A.; Gong, J. H.; Tam, E. M.; McCulloch, C. A.; Clark-Lewis, I.; Overall, C. M. Inflammation dampened by gelatinase A cleavage of monocyte chemoattractant protein-3. *Science* **2000**, *289*, 1202-1206. doi:10.1126/science.289.5482.1202
135. Rohani, M. G.; Parks, W. C. Matrix remodeling by MMPs during wound repair. *Matrix Biol* **2015**, *44-46*, 113-121. doi:10.1016/j.matbio.2015.03.002
136. Chinnathambi, S.; Bickenbach, J. R. Human skin and gingival keratinocytes show differential regulation of matrix metalloproteinases when combined with fibroblasts in 3-dimensional cultures. *J Periodontol* **2005**, *76*, 1072-1083. doi:10.1902/jop.2005.76.7.1072
137. Caley, M. P.; Martins, V. L.; O'Toole, E. A. Metalloproteinases and Wound Healing. *Adv Wound Care (New Rochelle)* **2015**, *4*, 225-234. doi:10.1089/wound.2014.0581
138. Krishnaswamy, V. R.; Mintz, D.; Sagi, I. Matrix metalloproteinases: The sculptors of chronic cutaneous wounds. *Biochim Biophys Acta Mol Cell Res* **2017**, *1864*, 2220-2227. doi:10.1016/j.bbamcr.2017.08.003
139. Letra, A.; Ghaneh, G.; Zhao, M.; Ray, H., Jr.; Francisconi, C. F.; Garlet, G. P.; Silva, R. M. MMP-7 and TIMP-1, new targets in predicting poor wound healing in apical periodontitis. *J Endod* **2013**, *39*, 1141-1146. doi:10.1016/j.joen.2013.06.015
140. Reiss, M. J.; Han, Y. P.; Garcia, E.; Goldberg, M.; Yu, H.; Garner, W. L. Matrix metalloproteinase-9 delays wound healing in a murine wound model. *Surgery* **2010**, *147*, 295-302. doi:10.1016/j.surg.2009.10.016
141. Kosten, I. J.; van de Ven, R.; Thon, M.; Gibbs, S.; de Gruijl, T. D. Comparative phenotypic and functional analysis of migratory dendritic cell subsets from human oral mucosa and skin. *PLoS One* **2017**, *12*, e0180333. doi:10.1371/journal.pone.0180333
142. Buskermolen, J. K.; Janus, M. M.; Roffel, S.; Krom, B. P.; Gibbs, S. Saliva-Derived Commensal and Pathogenic Biofilms in a Human Gingiva Model. *J Dent Res* **2018**, *97*, 201-208. doi:10.1177/0022034517729998
143. Haverman, T. M.; Laheij, A.; Nie, M.; Deng, D. M.; Raber-Durlacher, J. E.; de Soet, J. J.; Rozema, F. R. Exploring the role of oral microorganisms in the pathogenesis of mucositis by assessing their impact on metabolic activity and reproductive capacity of epithelial cells in vitro. *Support Care Cancer* **2020**, *28*, 4729-4735. doi:10.1007/s00520-020-05318-y
144. Laheij, A. M.; de Soet, J. J.; von dem Borne, P. A.; Kuijper, E. J.; Kraneveld, E. A.; van Loveren, C.; Raber-Durlacher, J. E. Oral bacteria and yeasts in relationship to oral ulcerations in hematopoietic stem cell transplant recipients. *Support Care Cancer* **2012**, *20*, 3231-3240. doi:10.1007/s00520-012-1463-2
145. Loomis, K. H.; Wu, S. K.; Ernlund, A.; Zudock, K.; Reno, A.; Blount, K.; Karig, D. K. A mixed community of skin microbiome representatives influences cutaneous processes more than individual members. *Microbiome* **2021**, *9*, 22. doi:10.1186/s40168-020-00963-1
146. Shapouri-Moghaddam, A.; Mohammadian, S.; Vazini, H.; Taghadosi, M.; Esmaeili, S. A.; Mardani, F.; Seifi, B.; Mohammadi, A.; Afshari, J. T.; Sahebkar, A. Macrophage plasticity, polarization, and function in health and disease. *J Cell Physiol* **2018**, *233*, 6425-6440. doi:10.1002/jcp.26429
147. Wynn, T. A. Fibrotic disease and the T(H)1/T(H)2 paradigm. *Nat Rev Immunol* **2004**, *4*, 583-594. doi:10.1038/nri1412
148. Vivier, E.; Artis, D.; Colonna, M.; Diefenbach, A.; Di Santo, J. P.; Eberl, G.; Koyasu, S.; Locksley, R. M.; McKenzie, A. N. J.; Mebius, R. E.; Powrie, F.; Spits, H. Innate Lymphoid Cells: 10 Years On. *Cell* **2018**, *174*, 1054-1066. doi:10.1016/j.cell.2018.07.017
149. van der Veer, W. M.; Bloemen, M. C.; Ulrich, M. M.; Molema, G.; van Zuijlen, P. P.; Middelkoop, E.; Niessen, F. B. Potential cellular and molecular causes of hypertrophic scar formation. *Burns* **2009**, *35*, 15-29. doi:10.1016/j.burns.2008.06.020
